# Comparative analysis of trends in the burden of motor neuron disease in China, the United States, and globally from 1990 to 2021: projections for 2022–2041

**DOI:** 10.3389/fneur.2025.1539889

**Published:** 2025-05-16

**Authors:** Bo Peng, Yuluo Tu, Cheng Zhou, Gui Xie, Jia Xiong, Kai Huang, Suifa Hu

**Affiliations:** Nanchang Hongdu Hospital of Traditional Chinese Medicine, Nanchang, China

**Keywords:** motor neuron disease, trend, incidence, mortality, prevalence, disability-adjusted life years

## Abstract

**Background:**

Motor Neuron Disease (MND) is a neurodegenerative disorder with low incidence (4–8 per 100,000), but high disability and mortality. This study analyzes MND burden in China, the U.S., and Globally from 1990 to 2021, covering trends in incidence, prevalence, mortality, Disability-Adjusted Life Years (DALYs), age-standardized incidence rate (ASIR), age-standardized prevalence rate (ASPR), age-standardized mortality rate (ASMR), and age-standardized DALYs rate (ASDR), and predicts changes for 2022–2041.

**Methods:**

Using Global Burden of Disease (GBD) data, Joinpoint regression analysis identified key turning points, and decomposition analysis quantified the contributions of aging, population growth, and epidemiological factors. The Autoregressive Integrated Moving Average (ARIMA) model forecasted future trends.

**Results:**

From 1990 to 2021, the incidence, prevalence, mortality, and DALYs of MND in China, the United States, and globally showed significant increases, with the largest increase observed in mortality (China +126%, USA +118%, global +156%). The annual average percentage changes (AAPC) were as follows: ASIR (−1.10, 0.45, −0.15%), ASPR (0.26, 0.39, −0.04%), ASMR (0.57, 0.51, 0.58%), and ASDR (−0.15, 0.14, 0.25%). Joinpoint regression analysis showed that China’s ASIR declined from 1990 to 2015 and began to rise in 2015, with ASPR continuing to increase and ASMR and ASDR fluctuating. In the US, ASIR continued to rise, ASPR decreased before 1995 and then increased, with fluctuations in ASMR and ASDR. Globally, ASIR and ASPR decreased from 1990 to 1995, increased from 1995 to 2005, and then declined from 2005 onwards, with ASMR continuing to increase. Decomposition analysis indicated that aging populations aged 65 and above were the primary driving factor. Gender and age analysis revealed that males under 69 bear a higher MND burden. Predictions for 2022–2041 show that in China, male ASIR will decline, while female ASIR will rise, with ASPR, ASMR, and ASDR remaining stable; in the US, male ASIR will rise, ASPR will first increase and then decrease, with ASMR and ASDR remaining stable; globally, ASIR, ASPR, and ASMR will remain stable, while male ASDR will rise and female ASDR will remain stable.

**Conclusion:**

The burden of MND continues to increase in China, the United States, and globally, with elderly males particularly affected, while low- and middle-income countries face more severe challenges. This study provides crucial data for developing global public health strategies and medical policies, aiming to effectively reduce the burden of MND through targeted interventions.

## Introduction

Motor Neuron Disease (MND) is a progressive neurodegenerative disorder characterized by the degeneration of motor neurons, leading to muscle weakness, paralysis, and eventual respiratory failure (1, 2). Subtypes such as Amyotrophic Lateral Sclerosis (ALS), Progressive Muscular Atrophy (PMA), and Primary Lateral Sclerosis (PLS) are associated with high disability and mortality rates (3). While MND is rare globally, with an annual prevalence of 4–8 per 100,000, its impact is disproportionately high due to its rapid progression, lack of curative treatments, and significant care requirements (4, 5). The increasing burden of MND poses a growing public health challenge, especially in aging societies (6).

Over the past three decades, the global burden of MND has risen significantly, driven primarily by demographic shifts such as population aging and growth, alongside improvements in diagnostic capabilities (7). These changes have led to higher prevalence and longer survival among MND patients, amplifying the disease’s cumulative impact on healthcare systems (4, 5, 7). Countries like China and the United States, which are experiencing rapid demographic transitions, are particularly affected (8, 9). Understanding the long-term trends and drivers of MND burden is critical for informing healthcare policies and resource allocation. Despite advances in understanding the pathophysiology and treatment of MND (1, 10, 11), there remain substantial gaps in knowledge about the disease’s epidemiological trends and future projections. Existing research often focuses on localized data or limited timeframes (8, 9), neglecting the broader global perspective and the interplay of demographic and epidemiological factors. Moreover, few studies have incorporated predictive analyses to anticipate future trends, leaving healthcare systems unprepared for the rising demand associated with MND care. Addressing these gaps requires comprehensive and longitudinal analyses that integrate historical data with robust forecasts.

The 2021 Global Burden of Disease (GBD) study is a publicly accessible database offering a comprehensive repository of data on the burden of 371 diseases and injuries worldwide (12). This study utilizes the 2021 GBD database to comprehensively evaluate the burden of MND in China, the United States, and globally from 1990 to 2021, focusing on incident cases, prevalence, deaths, Disability-Adjusted Life Years (DALYs), as well as age-standardized incidence rate (ASIR), age-standardized prevalence rate (ASPR), age-standardized mortality rate (ASMR), and age-standardized DALYs rate (ASDR). To identify key trend changes, Joinpoint regression analysis was applied to calculate the annual average percentage changes (AAPC) for each metric. Furthermore, decomposition analysis quantified the contributions of aging, population growth, and epidemiological factors to observed changes in MND burden. Using Autoregressive Integrated Moving Average (ARIMA) models, the study forecasts trends in MND burden for China, the United States, and globally from 2022 to 2041, providing insights to inform targeted prevention strategies and ensure equitable allocation of public health resources.

## Methods

### Data source

The data used in this study were extracted from the GBD 2021 datasets, a comprehensive database documenting the burden of 371 diseases and injuries and 88 risk factors across 204 countries and regions (12). The data are categorized by age and gender to enable detailed analyses. The GBD datasets integrates information from multiple sources, including cohort studies, randomized controlled trials, national health surveys, hospital records, vital statistics registries, and other research, providing reliable and consistent estimates of disease burden (13–15). This study utilized the Global Health Data Exchange (GHDx) online database (http://ghdx.healthdata.org) and the GHDx query tool (http://ghdx.healthdata.org/gbd-results-tool) to extract data for MND.The metrics analyzed included incidence, prevalence, mortality, and DALYs for 19 age groups(from <5 to ≥95 years at 5-year intervals), stratified by gender (both sexes, male and female) for China, the United States, and globally from 1990 to 2021.MND is defined as a group of progressive neurodegenerative disorders affecting motor neurons in the brain and spinal cord (1, 2). The main subtypes include ALS, PMA, and PLS. In this study, MND diagnosis codes were based on ICD-10 codes G12.2 and G12.8, as well as ICD-9 codes 335.20, 335.21, and 335.24.

### Study design

This population-based retrospective study analyzed the burden of MND using data extracted from the 2021 GBD database. The analysis covered the period from 1990 to 2021, focusing on three geographic regions: China, the United States, and globally. The primary objective was to evaluate trends in MND burden and project future trends from 2022 to 2041, providing insights to inform healthcare planning and targeted interventions. Key metrics analyzed included incidence, prevalence, mortality, DALYs, ASIR, ASPR, ASMR, and ASDR.

## Statistical analysis

### Descriptive and temporal analysis

The burden of MND was assessed using data on incidence, prevalence, mortality, and DALYs. Crude and age-standardized rates, including incidence, prevalence, mortality, and DALYs, were analyzed for China, the United States, and globally from 1990 to 2021. Age standardization was performed using the GBD standard population, which involves applying a weighted average of age-specific rates based on a reference population’s age distribution. This method adjusts for differences in age structure across populations, allowing for more accurate comparisons both over time and across regions. By employing this approach, the analysis controlled for demographic differences, enabling meaningful comparisons of MND burden trends across different time periods and populations, while ensuring the reliability and consistency of the results.

### Confidence intervals (CI) and uncertainty intervals (UI)

CI and UI are both used to express the range of values within which a parameter is expected to lie, but they differ in their context and application. CI typically represent the range within which we expect the true population parameter to lie, based on sample data, with a specified level of confidence (usually 95%). CI are commonly used to indicate the precision of statistical estimates, such as the AAPC in trend analysis, and reflect the variability due to sampling error. In contrast, UI are used in the context of GBD studies and account for a broader range of uncertainties, including data quality, model assumptions, and estimation processes. UI provide a range of possible values reflecting the potential variability in disease burden estimates under different assumptions and are typically reported as the 95% UI to capture the uncertainty inherent in the GBD model. Therefore, while CI focus on the precision of estimates from sample data, UI reflect the overall uncertainty in model-based estimates, making them a key tool in GBD analyses.

### Joinpoint regression analysis

To determine the burden trend of MND, this study used Joinpoint software (National Cancer Institute, Rockville, MD, USA) to calculate the AAPC and its corresponding 95% CI (16, 17). In this process, logarithmic age-standardized indicators were fitted into a regression model: ln(y) = *α* + *β*x + *ε*, where y represents the respective age-standardized indicator, and x represents the calendar year. The AAPC was calculated as 100 × (exp(β) − 1), and the 95% CI was also derived from the model. If the 95% CI of the corresponding AAPC estimate is greater than 0, it indicates an increasing trend in the age-standardized indicator; if less than 0, it indicates a decreasing trend; and if it includes 0, it indicates a stable trend. Additionally, Joinpoint regression analysis can identify “change points” in the time series, where significant shifts in trends occur, providing precise time points for changes in the MND burden trend. This method effectively controls for demographic differences, enabling detailed comparisons of MND burden trends across different regions and time periods, ensuring the reliability and scientific rigor of the results.

### Decomposition analysis

This study used decomposition analysis to quantify the contributions of population aging, epidemiological changes, and population growth to the changes in the burden of MND (17). The method employed the standard decomposition approach based on the Lee-Carter model, which decomposes the changes in MND burden into three main factors: population aging, epidemiological changes, and population growth. Specifically, the population aging effect measures how the increase in the elderly population due to changes in the age structure contributes to the rise in MND burden; the epidemiological change effect analyzes the changes in age-specific disease rates, reflecting the impact of improvements in medical care, treatment, or disease prevention on the burden; and the population growth effect-quantifies the impact of total population growth on the MND burden, with the understanding that even if age-specific disease rates remain unchanged, population growth will still lead to an increased disease burden. By using the Lee-Carter model, we were able to independently assess the contributions of these three factors to the changes in MND burden and distinguish their different impacts on disease trends. This analysis helps identify the key drivers of changes in MND burden and provides evidence for the formulation of more targeted public health policies.

### Predictive analysis

Future trends in MND burden from 2022 to 2041 were forecasted using ARIMA models (18). Historical data from 1990 to 2021, including incidence, prevalence, mortality, and DALYs, were used to train the model. The data were tested for stationarity, with differencing applied as needed. The optimal ARIMA model parameters were determined using Akaike Information Criterion and Bayesian Information Criterion, with diagnostic checks for autocorrelation. K-fold cross-validation was performed to assess accuracy, using root mean squared error and mean absolute percentage error to quantify prediction error. Forecasts for 2022–2041 were generated and evaluated against historical trends and literature for reliability. Residual diagnostics confirmed the assumptions of linearity, stationarity, and no residual autocorrelation. These rigorous methods ensured reliable projections of future MND burden trends for public health planning and policy.

### Data visualization

Statistical analysis and visualization were performed using R (Version 4.4.1) and Python (Version 3.9). Joinpoint regression results were visualized to illustrate trend shifts and AAPC estimates, while Tableau (Version 2021.4) was used to create figures summarizing global and regional MND burden trends.

### Ethical considerations

This study relied solely on publicly available, de-identified data from the GBD database, ensuring compliance with ethical guidelines. Since no human participants were involved, ethical approval was not required. The study adhered to the principles outlined in the Declaration of Helsinki, ensuring scientific integrity and transparency.

## Results

### Description of the burden of MND in China, the United States, and globally

#### Incidence of MND in China, the United States, and globally

In China, the number of newly diagnosed MND cases increased from 6,854 in 1990 [95% UI: 5,928–7,956] to 7,324 in 2021 (95% UI: 5,993–8,709), a cumulative increase of 6.86%. The ASIR decreased from 0.65 per 100,000 (95% UI: 0.57–0.75) in 1990 to 0.46 per 100,000 (95% UI: 0.39–0.54) in 2021, with an AAPC in incidence rate of-1.10% (95% CI: −1.22 to −0.99). In the United States, the number of new MND cases increased from 5,281 in 1990 (95% UI: 5,004–5,604) to 11,089 in 2021 (95% UI: 10,634–11,559), a cumulative increase of 109.98%. ASIR increased from 1.79 per 100,000 (95% UI: 1.69–1.91) in 1990 to 2.06 per 100,000 (95% UI: 2.01–2.11) in 2021, with an AAPC increase of 0.45% (95% CI: 0.42–0.48). Globally, the number of new MND cases increased from 36,769 cases (95% UI: 33,068–41,300) in 1990 to 64,178 cases (95% UI: 58,506–70,270) in 2021, a cumulative increase of 74.54%. The global ASIR decreased from 0.81 cases per 100,000 (95% UI: 0.72–0.90) in 1990 to 0.77 cases per 100,000 (95% UI: 0.70–0.84) in 2021, with an AAPC decrease of 0.15% (95% CI: −0.18–-0.12) (Table 1).

**Table 1:** All-age cases and age-standardized incidence, prevalence, mortality, and DALYs rates and corresponding AAPC of MND in China, the United States, and Globally in 1990 and 2021.

Location	Sex name	Measure	1990		2021		1990–2021
			All-ages cases	Age-standardized rates per 100,000 people	All-ages cases	Age-standardized rates per 100,000 people	AAPC
			*n* (95%UI)	*n* (95% UI)	*n* (95%UI)	*n* (95% UI)	*n* (95% CI)
China	Both	Deaths	1,528 (845, 1953)	0.15 (0.09, 0.19)	3,450 (2,220, 4,790)	0.18 (0.11, 0.25)	0.57* (0.26, 0.88)
		DAL Ys	87,564 (50,251, 111,668)	7.99 (4.67, 10.15)	122,661 (81,013, 167,332)	7.67 (4.88, 10.06)	−0.15 (−0.51, 0.22)
		Prevalence	25,691 (20,344, 31,960)	2.13 (1.72, 2.60)	33,342 (27,028, 40,366)	2.30 (1.84, 2.80)	0.26* (0.21, 0.30)
		Incidence	6,854 (5,928, 7,956)	0.65 (0.57, 0.75)	7,324 (5,993, 8,709)	0.46 (0.39, 0.54)	−1.10* (−1.22, −0.99)
	Male	Deaths	802 (86, 1,189)	0.15 (0.02, 0.23)	1939 (667, 3,131)	0.21 (0.07, 0.33)	0.95* (0.56, 1.34)
		DALYs	45,779 (7,256, 66,689)	7.87 (1.22, 11.46)	70,894 (27,361, 113,107)	8.94 (3.44, 13.64)	0.38 (−0.01, 0.77)
		Prevalence	13,281 (10,533, 16,576)	2.13 (1.72, 2.62)	16,980 (13,774, 20,513)	2.35 (1.86, 2.83)	0.32* (0.28, 0.36)
		Incidence	3,667 (3,165, 4,241)	0.67 (0.58, 0.78)	3,932 (3,269, 4,636)	0.50 (0.43, 0.57)	−0.95* (−0.87, −1.02)
	Female	Deaths	726 (609, 841)	0.15 (0.13, 0.17)	1,511 (1,187, 1,912)	0.15 (0.12, 0.19)	0.04 (−0.37, 0.44)
		DALYs	41,785 (36,355, 48,767)	8.20 (7.13, 9.58)	51,767 (41,917, 64,348)	6.30 (5.13, 7.67)	−0.85* (−1.24, −0.46)
		Prevalence	12,410 (9,852, 15,429)	2.13 (1.72,2.61)	16,362 (13,112, 19,981)	2.25 (1.79, 2.75)	0.18* (0.13, 0.24)
		Incidence	3,187 (2,758, 3,675)	0.63 (0.55, 0.72)	3,392 (2,769, 4,072)	0.42 (0.35, 0.50)	−1.27* (−1.42, −1.13)
the United States	Both	Deaths	3,890 (3,687, 4,008)	1.26 (1.20, 1.29)	8,465 (7,800, 8,899)	1.49 (1.38, 1.56)	0.51* (0.12, 0.91)
		DALYs	111,652 (107,616, 114,636)	39.71 (38.48, 40.72)	210,559 (199,250, 218,997)	41.36 (39.47, 42.94)	0.14 (−0.22, 0.50)
		Prevalence	22,197 (19,257, 25,430)	7.82 (6.82, 8.92)	40,495 (37,645, 43,576)	8.82 (8.26, 9.51)	0.39* (0.29, 0.48)
		Incidence	5,281 (5,004, 5,604)	1.79 (1.69, 1.91)	11,089 (10,634, 11,559)	2.06 (1.98, 2.14)	0.45* (0.42, 0.48)
	Male	Deaths	2035 (1963, 2087)	1.52 (1.46, 1.56)	4,705 (4,441, 4,912)	1.80 (1.71, 1.88)	0.39* (0.07, 0.72)
		DALYs	60,845 (59,152, 62,402)	46.99 (45.72, 48.22)	120,100 (114,053, 125,007)	50.01 (47.66, 51.99)	0.09 (−0.15, 0.34)
		Prevalence	12,069 (10,460, 13,881)	9.21 (8.01, 10.55)	22,755 (21,263, 24,480)	10.33 (9.74, 11.13)	0.37* (0.29, 0.45)
		Incidence	2,804 (2,653, 2,980)	2.13 (2.01, 2.26)	6,154 (5,915, 6,410)	2.46 (2.37, 2.57)	0.48* (0.44, 0.51)
	Female	Deaths	1855 (1724, 1928)	1.06 (1.00, 1.10)	3,760 (3,366, 4,020)	1.21 (1.10, 1.29)	0.44 (−0.10, 0.99)
		DALYs	50,807 (48,449, 52,383)	33.49 (32.28, 34.43)	90,459 (83,918, 95,384)	33.45 (31.52, 34.99)	−0.01 (−0.43, 0.42)
		Prevalence	10,128 (8,779, 11,581)	6.62 (5.74, 7.55)	17,740 (16,414, 19,155)	7.46 (6.96, 8.05)	0.37* (0.24, 0.51)
		Incidence	2,477 (2,339, 2,627)	1.52 (1.43, 1.62)	4,935 (4,719, 5,152)	1.70 (1.63,1.78)	0.36* (0.33, 0.40)
Global	Both	Deaths	15,260 (14,367, 16,043)	0.38 (0.36, 0.40)	39,082 (35,757, 42,433)	0.46 (0.42, 0.49)	0.58* (0.45, 0.70)
		DALYs	506,146 (462,035–545,050)	11.22 (10.39, 11.98)	1,040,566 (963,064, 1,123,956)	12.17 (11.24, 13.15)	0.25* (0.09, 0.42)
		Prevalence	161,926 (137,006, 189,263)	3.36 (2.87, 3.92)	272,732 (236,194, 313,676)	3.31 (2.86, 3.80)	−0.04 (−0.15, 0.06)
		Incidence	36,769 (33,068, 41,300)	0.81 (0.72,0.90)	64,178 (58,506, 70,270)	0.77 (0.70, 0.84)	−0.15* (−0.18, −0.12)
	Male	Deaths	8,012 (7,137, 8,579)	0.44 (0.40, 0.46)	21,703 (19,623,23,371)	0.55 (0.50, 0.59)	0.72* (0.54, 0.91)
		DALYs	275,249 (230,289, 305,186)	12.73 (11.00, 13.82)	592,049 (529,600, 642,811)	14.45 (12.93, 15.76)	0.41* (0.19, 0.62)
		Prevalence	84,544 (98,825, 71,637)	3.61 (3.11, 4.21)	145,479 (126,364, 166,787)	3.64 (3.16, 4.16)	0.04 (−0.07, 0.15)
		Incidence	19,685 (17,693, 22,183)	0.90 (0.81,1.00)	35,138 (32,151, 38,498)	0.88 (0.81,0.96)	−0.06* (−0.09, −0.03)
	Female	Deaths	7,248 (6,819, 7,577)	0.33 (0.31, 0.35)	17,379 (15,419, 19,638)	0.38 (0.34, 0.43)	0.38* (0.21, 0.55)
		DALYs	230,897 (219,091, 243,419)	9.86 (9.38, 10.36)	448,517 (413,228, 496,721)	10.08 (9.33, 11.14)	0.06 (−0.13, 0.25)
		Prevalence	77,382 (65,287, 90,853)	3.13 (2.66, 3.66)	127,253 (109,471, 146,510)	3.02 (2.60, 3.52)	−0.12* (−0.21, −0.02)
		Incidence	17,084 (15,300, 19,087)	0.72 (0.65, 0.81)	29,040 (26,390, 31,866)	0.67(0.61, 0.74)	−0.25*(−0.29, −0.21)

#### Prevalence of MND in China, the United States, and globally

In China, the number of MND patients increased from 25,691 in 1990 (95% UI: 20,344–31,960) to 33,342 in 2021 (95% UI: 27,028–40,366), a cumulative increase of 29.78%. The ASPR increased from 2.13 per 100,000 (95% UI: 1.72–2.60) in 1990 to 2.30 per 100,000 (95% UI: 1.84–2.80) in 2021, with an AAPC increase of 0.26% (95% CI: 0.21–0.30). In the United States, the number of MND patients increased from 22,197 in 1990 (95% UI: 19,257–25,430) to 40,495 in 2021 (95% UI: 37,645–43,576), a cumulative increase of 82.43%. The ASPR increased from 7.82 per 100,000 (95% UI: 6.82–8.92) in 1990 to 8.82 per 100,000 (95% UI: 8.26–9.51) in 2021, with an AAPC increase of 0.39% (95% CI: 0.29–0.48). Globally, the number of MND patients increased from 161,926 in 1990 (95% UI: 137,006–189,263) to 272,732 in 2021 (95% UI: 236,194–313,676), a cumulative increase of 68.43%. The ASPR decreased from 3.36 per 100,000 (95% UI: 2.87–3.92) in 1990 to 3.31 per 100,000 (95% UI: 2.86–3.80) in 2021, with an AAPC decrease of −0.04% (95% CI: −0.15 to 0.06), which was not statistically significant. This indicates that, although a slight decrease in the absolute value was observed, the global prevalence trend did not show a significant change (Table 1).

#### Mortality of MND in China, the United States, and globally

In China, the number of MND-related deaths increased from 1,528 in 1990 (95% UI: 845–1,953) to 3,450 in 2021 (95% UI: 2,220–4,790), a cumulative increase of 125.79%. The ASMR increased from 0.15 per 100,000 (95% UI: 0.09–0.19) in 1990 to 0.18 per 100,000 (95% UI: 0.11–0.25) in 2021, with an AAPC increase of 0.57% (95% CI: 0.26–0.88). In the United States, the number of MND-related deaths increased from 3,890 in 1990 (95% UI: 3,687–4,008) to 8,465 in 2021 (95% UI: 7,800–8,899), a cumulative increase of 117.60%. The ASMR increased from 1.26 per 100,000 (95% UI: 1.20–1.29) in 1990 to 1.49 per 100,000 (95% UI: 1.38–1.56) in 2021, with an AAPC increase of 0.51% (95% CI: 0.12–0.91). Globally, the number of MND-related deaths increased from 15,260 in 1990 (95% UI: 14,367–16,043) to 39,082 in 2021 (95% UI: 35,757–42,433), a cumulative increase of 156.11%. The ASMR increased from 0.38 per 100,000 (95% UI: 0.36–0.40) in 1990 to 0.46 per 100,000 (95% UI: 0.42–0.49) in 2021, with an AAPC increase of 0.58% (95% CI: 0.45–0.70) (Table 1).

#### DALYs of MND in China, the United States, and globally

In China, the DALYs related to MND were 87,564 in 1990 (95% UI: 50,251–111,668) and increased to 122,661 in 2021 (95% UI: 81,013–167,332), a growth of 40.08%. The Age-Standardized DALY Rate (ASDR) decreased from 7.99 per 100,000 (95% UI: 4.67–10.15) in 1990 to 7.67 per 100,000 (95% UI: 4.88–10.06) in 2021, with an AAPC decrease of −0.15% (95% CI: −0.51–0.22). In the United States, MND-related DALYs were 111,652 in 1990 (95% UI: 107,616–114,636) and increased to 210,559 in 2021 (95% UI: 199,250–218,997), a growth of 88.59%. The ASDR increased from 39.71 per 100,000 (95% UI: 38.48–40.72) in 1990 to 41.36 per 100,000 (95% UI: 39.47–42.94) in 2021, with an AAPC increase of 0.14% (95% CI: −0.22–0.50). Globally, MND-related DALYs increased from 506,146 in 1990 (95% UI: 462,035–545,050) to 1,040,566 in 2021 (95% UI: 963,064–1,123,956), a growth of 105.58%. The ASDR increased from 11.22 per 100,000 (95% UI: 10.39–11.98) in 1990 to 12.17 per 100,000 (95% UI: 11.24–13.15) in 2021, with an AAPC increase of 0.25% (95% CI: 0.09–0.42) (Table 1).

#### Joinpoint regression analysis of the burden of MND in China, the United States, and globally

**Figure 1:**
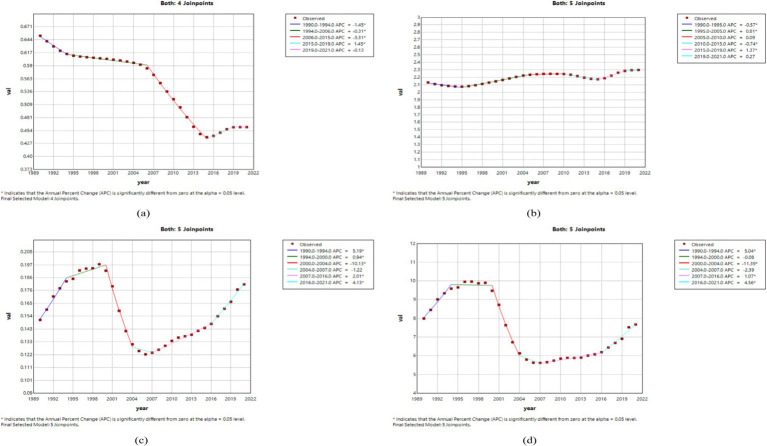
The APC of ASIR, ASPR, ASMR, and ASDR of MND in China from 1990 to 2021 (*means *p*-values<0.05 and significant results). **(A)** ASIR; **(B)** ASPR; **(C)** ASMR; **(D)** ASDR.

**Figure 2:**
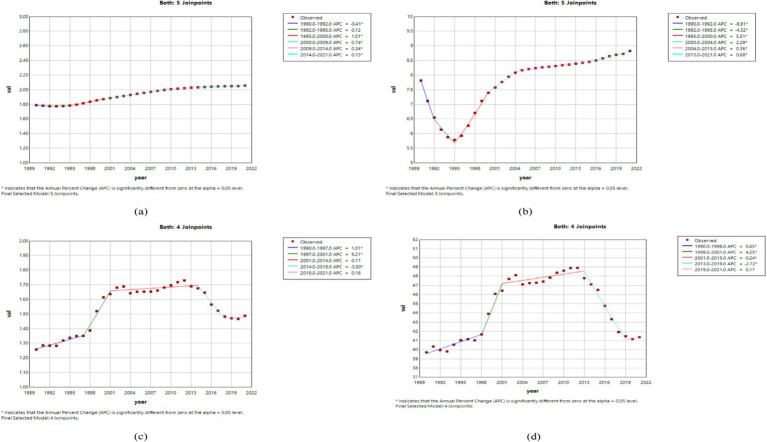
The APC of ASIR, ASPR, ASMR, and ASDR of MND in the United States from 1990 to 2021 (*means *p*-values<0.05 and significant results). **(A)** ASIR; **(B)** ASPR; **(C)** ASMR; **(D)** ASDR.

**Figure 3:**
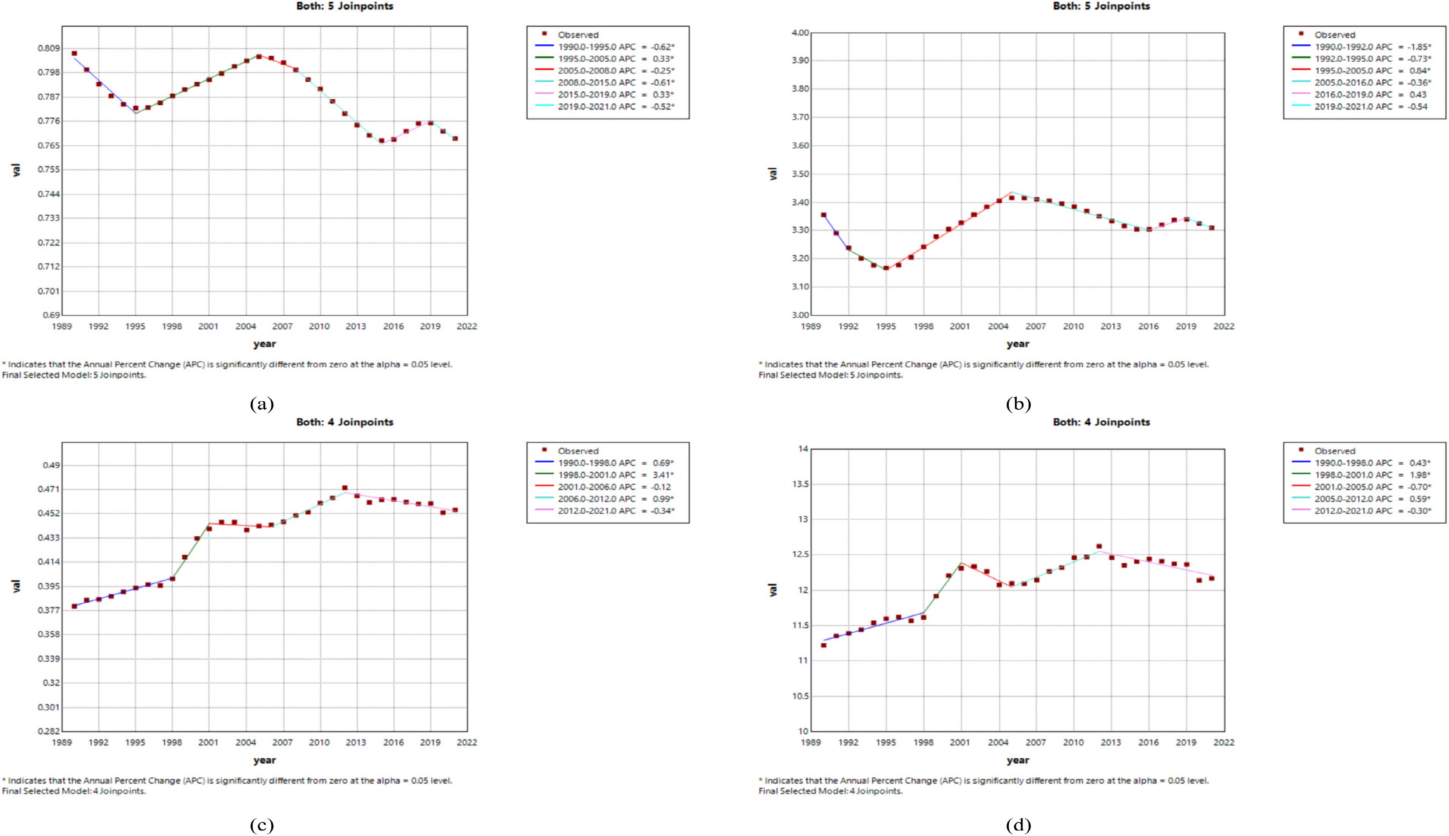
The APC of ASIR, ASPR, ASMR, and ASDR of MND in Global from 1990 to 2021 (*means *p*-values<0.05 and significant results). **(A)** ASIR; **(B)** ASPR; **(C)** ASMR; **(D)** ASDR.

The results of the Joinpoint regression analysis from 1990 to 2021 revealed notable temporal changes and regional heterogeneity in the four age-standardized indicators of MND–ASIR, ASPR, ASMR, and ASDR–across China, the United States, and the global level. Overall, ASIR in China showed a consistent decline before 2015, followed by an upward trend thereafter, while ASPR demonstrated a steady increase beginning in 1995. In the United States, ASIR gradually increased from the early 1990s, and ASPR rebounded sharply after an initial decline, then stabilized. Globally, both ASIR and ASPR declined during 1990–1995, rose between 1995 and 2005, and declined again after 2005. ASMR and ASDR in China and the United States exhibited a stage-like “increase–decrease–increase” fluctuation, while globally they showed a relatively stable but cyclically increasing trend. Specifically, in China, ASIR decreased significantly from 1990 to 2015 (APC = −1.45 for 1990–1994, −0.31 for 1994–2006, and −3.31 for 2006–2015; all *p* < 0.05), increased notably from 2015 to 2019 (APC = 1.45, *p* < 0.05), and then slightly declined after 2019 (Figure 1A). ASPR experienced a brief decline from 1990 to 1995 (APC = −0.57, *p* < 0.05), followed by a continuous rise, with significant increases during 1995–2005 (APC = 0.81, *p* < 0.05) and 2015–2019 (APC = 1.37, *p* < 0.05) (Figure 1B). ASMR and ASDR in China showed a fluctuating pattern, with ASMR increasing rapidly during 1990–1994 (APC = 5.19, *p* < 0.05), declining during 2000–2004 (APC = −10.13, *p* < 0.05), and rising again from 2016 to 2021 (APC = 4.13, *p* < 0.05), with ASDR showing similar trends (Figures 1C,D). In the United States, ASIR slightly declined between 1990 and 1992 (APC = −0.41, *p* < 0.05), then continued to increase, particularly during 1995–2000 (APC = 1.01, *p* < 0.05) and 2014–2021 (APC = 0.15, *p* < 0.05) (Figure 2A). ASPR showed a marked decrease from 1990 to 1995 (APC = −8.91 for 1990–1992, *p* < 0.05), but rebounded strongly during 1995–2004 (APC = 5.51 for 1995–2000, 2.29 for 2000–2004; both *p* < 0.05), with the growth rate slowing after 2004 (Figure 2B). ASMR and ASDR both demonstrated upward fluctuations, with ASMR rising significantly from 1997 to 2001 (APC = 5.21, *p* < 0.05), then declining during 2014–2018 (APC = −3.50, *p* < 0.05) (Figures 2C,D). Globally, ASIR and ASPR both declined from 1990 to 1995 (ASIR: APC = −0.62, p < 0.05; ASPR: APC = −1.85 for 1990–1992, −0.73 for 1992–1995; both *p* < 0.05), increased during 1995–2005 (ASIR: APC = 0.33; ASPR: APC = 0.84; both *p* < 0.05), and then generally declined after 2005 (Figures 3A,B). Although ASMR and ASDR showed an overall upward trend globally, there were notable fluctuations. For example, ASMR increased significantly from 1998 to 2001 (APC = 3.41, *p* < 0.05) and declined from 2012 to 2021 (APC = −0.34, *p* < 0.05), while ASDR increased from 2005 to 2012 (APC = 0.59, *p* < 0.05), then turned downward in the subsequent years (Figures 3C,D).

#### Burden of MND disease in different age groups in China, the United States, and globally in 1990 and 2021

**Figure 4:**
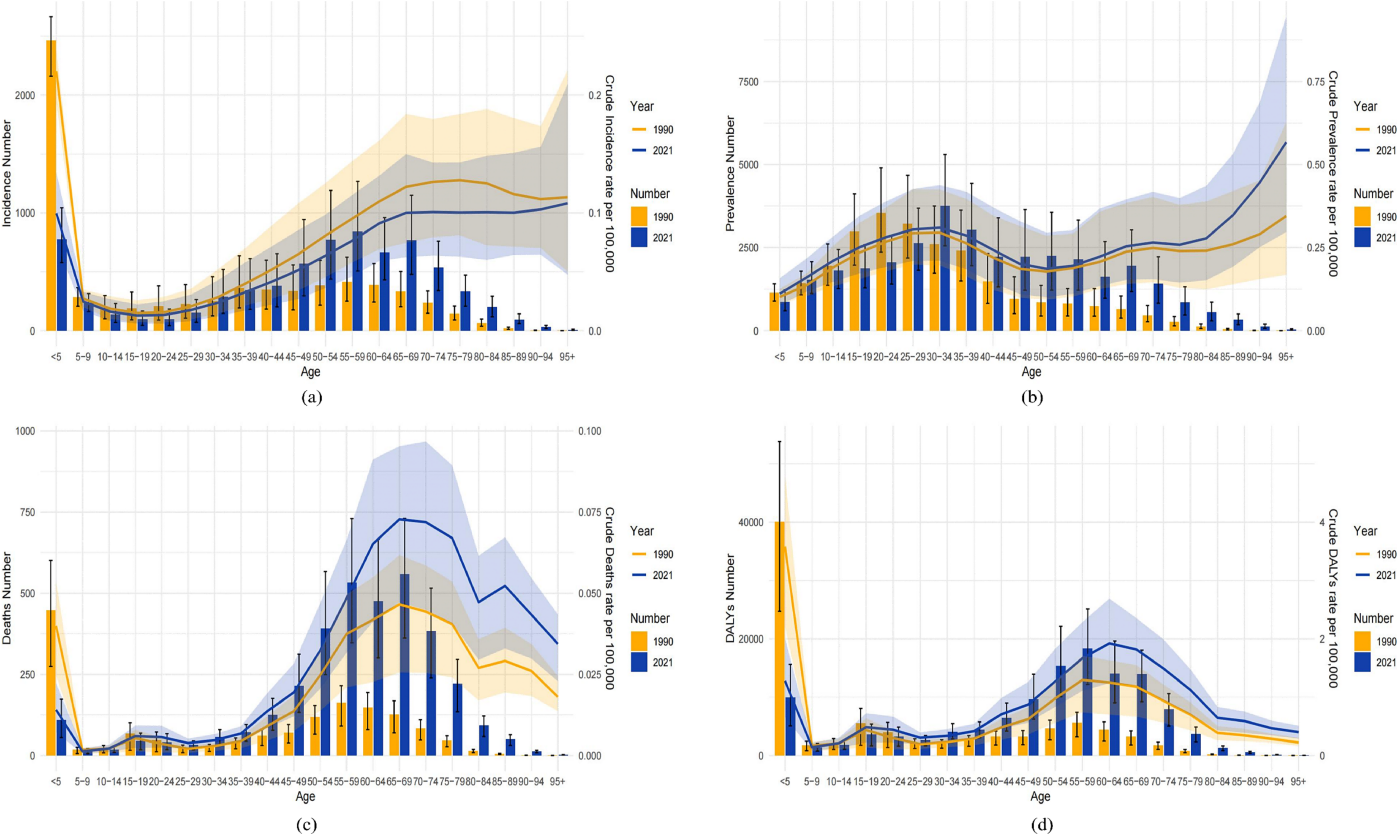
Comparative of the incidence, prevalence, deaths, and DALYs counts, along with their crude rates, by age group in China from 1990 and 2021. **(A)** Incident cases and CIR; **(B)** Prevalent cases and CPR; **(C)** Death cases and CMR; **(D)** DALYs counts and CDR; Bar charts represent counts; lines represent crude rates.

By analyzing the incidence, prevalence, mortality, and DALYs of MND in China, the United States, and Globally for different age groups in 1990 and 2021, this study reveals the trends and changes in the disease burden and crude rates across different regions and time periods. In China, the incidence of MND in 1990 and 2021 was concentrated in the 0–5 and 45–74 age groups. The crude incidence rate (CIR) showed a declining trend in the 0–14 age group, but an increasing trend in the 20–69 age groups (Figure 4A). In terms of prevalence, the peak number of cases in both 1990 and 2021 occurred in the 15–44 age group. The crude prevalence rate (CPR) increased in the 0–34 age group, decreased in the 35–54 age group, and then increased again after the age of 55 (Figure 4B). For mortality, the peak number of deaths in 1990 occurred in the 0–5 age group, while in 2021, it shifted to the 65–69 age group. The crude mortality rate (CMR) showed a declining trend in the 0–9 age group but an increasing trend in the 10–69 age groups (Figure 4C). In terms of DALYs, the peak DALYs in 1990 occurred in the 0–5 age group, while in 2021, it shifted to the 55–59 age group. The crude DALY rate (CDR) decreased in the 0–9 age group and increased in the 10–64 age group (Figure 4D). In the United States, the peak incidence and prevalence of MND in both 1990 and 2021 occurred in the 65–74 age group. The CIR showed a declining trend in the 0–9 age group, an increasing trend in the 10–79 age group, and then a declining trend after 80 years of age (Supplementary Figure 1A). For prevalence, the CPR increased in the 0–79 age groups, but decreased after 80 years of age (Supplementary Figure 1B). In terms of mortality, the peak mortality for MND in both 1990 and 2021 occurred in the 70–74 age group, with the CMR showing an increasing trend in the 5–79 age group and a decreasing trend after 80 years of age (Supplementary Figure 1C). Regarding DALYs, the age group with the highest DALYs in both 1990 and 2021 was 60–74 years, with the peak DALYs in 1990 occurring in the 65–74 age group, while in 2021, the peak shifted to the 75–79 age group (Supplementary Figure 1D). Globally, the highest incidence of MND in 1990 occurred in the 0–5 age group, but by 2021, it shifted to the 65–69 age group. The CIR showed a declining trend in the 0–9 age group, with an increasing trend in the 10–79 age group (Supplementary Figure 2A). In terms of prevalence, the peak number of cases in 1990 occurred in the 20–24 age group, but in 2021, it shifted to the 70–74 age group. The CPR showed a gradual increase across the 0–79 age groups, with the peak occurring in the 75–79 age group (Supplementary Figure 2B). For mortality and DALYs, the peak death toll and DALYs in both 1990 and 2021 were concentrated in the 55–79 age group, with the CMR and CDR being lower before the age of 40 and gradually increasing after that age (Supplementary Figures 2C,D).

#### Gender disparities in the burden of MND in different age groups in China, the United States, and globally

**Figure 5:**
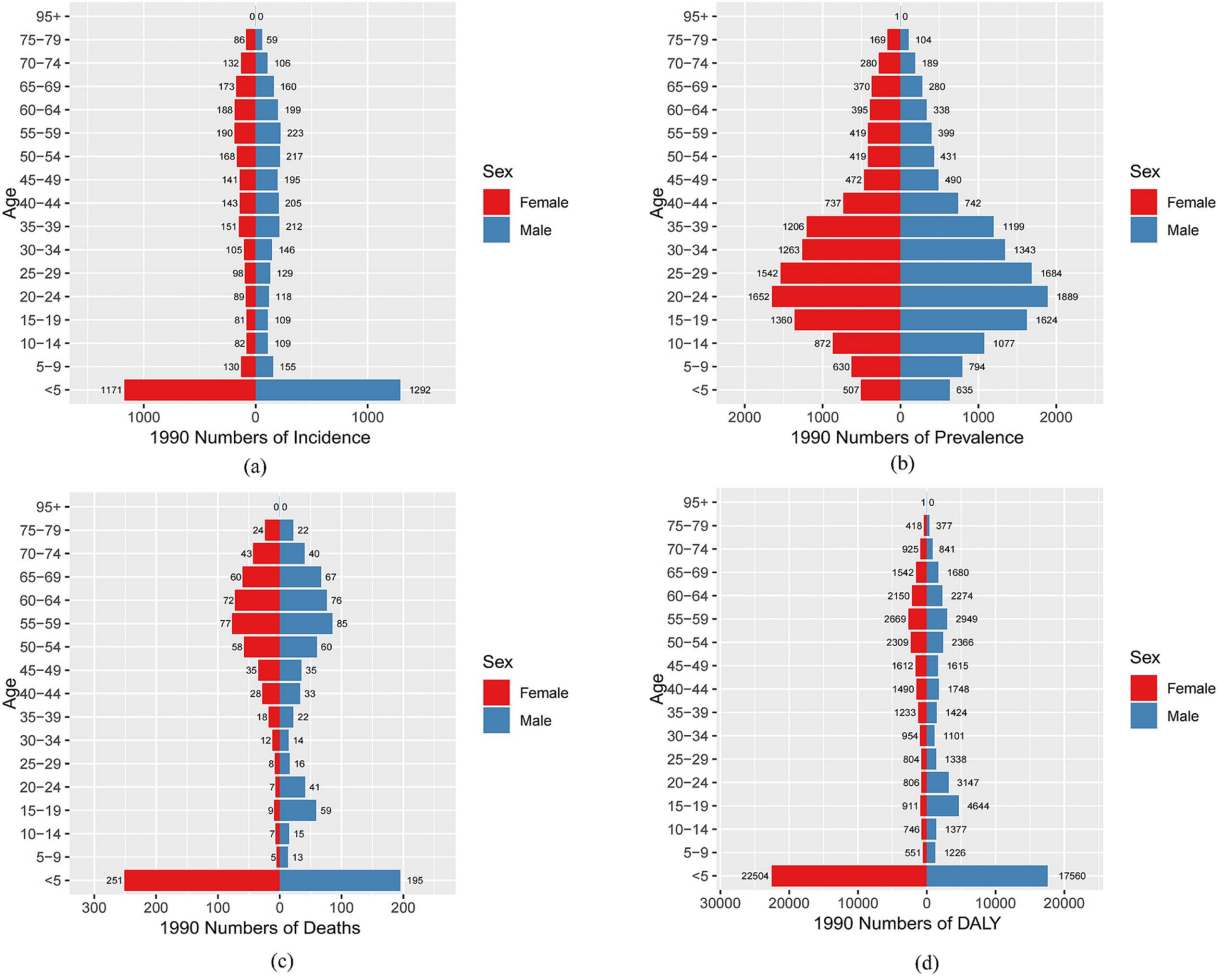
Comparison of the number of incidence, prevalence, mortality, and DALYs of MND in males and females of different age groups in China in 1990. **(A)** Incidence; **(B)** Prevalence; **(C)** Mortality; **(D)** DALYs.

**Figure 6:**
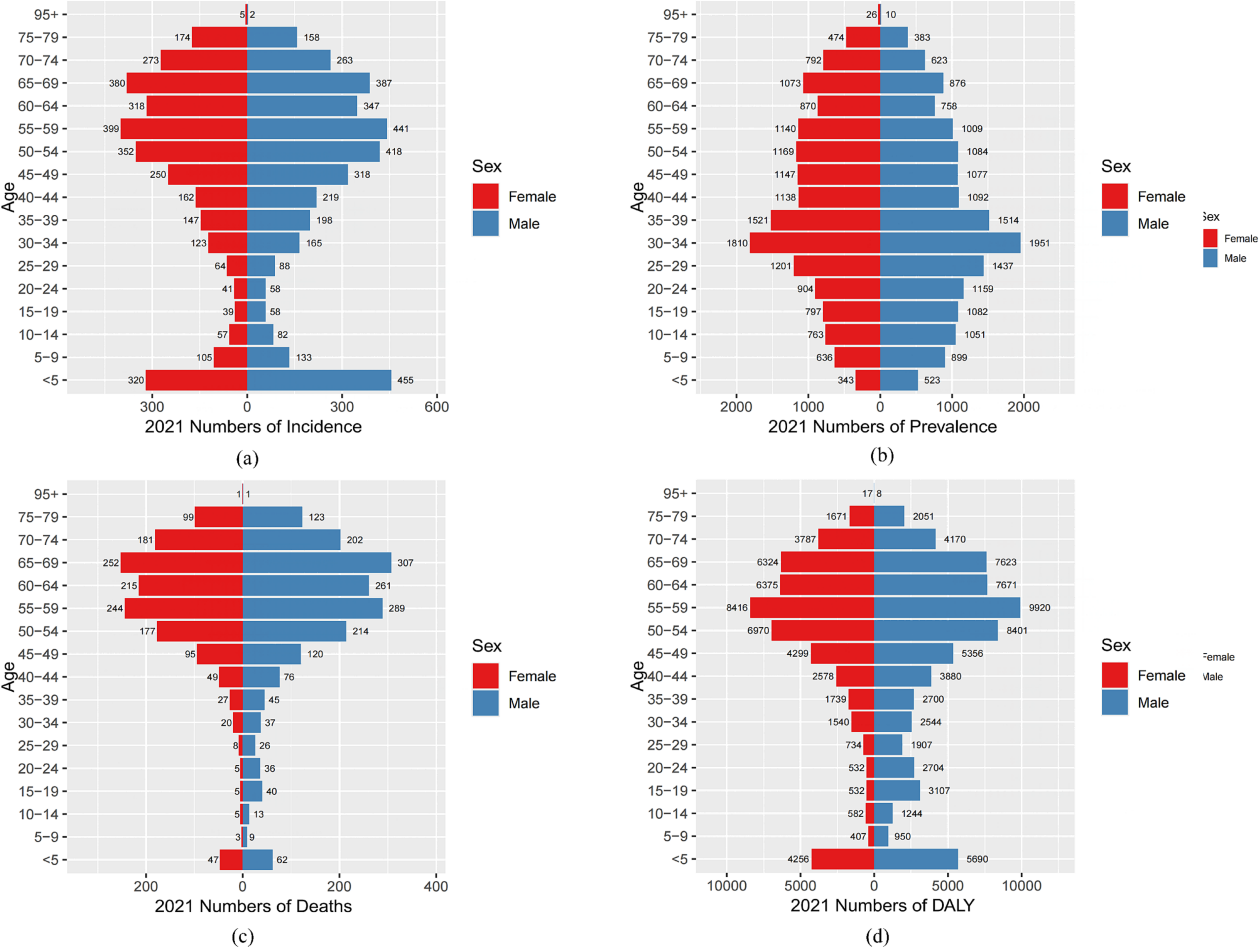
Comparison of the number of incidence, prevalence, mortality, and DALYs of MND in males and females of different age groups in China in 2021. **(A)** Incidence; **(B)** Prevalence; **(C)** Mortality; **(D)** DALYs.

Figures 5, 6 and Supplementary Figures 3–6, respectively, present the incidence, prevalence, mortality, and DALYs of MND among males and females across different age groups in China, the United States, and globally in 1990 and 2021. In 1990, the peak incidence of MND in China and globally for both males and females occurred in the 0–5 age group (Figure 5A and Supplementary Figure 5A), whereas the United States showed a different trend, with the peak incidence for males in the 65–69 age group and for females in the 70–74 age group (Supplementary Figure 3A). In all age groups, the incidence of MND was higher in males than in females in China for those under 64 years of age (Figure 5A), and in the United States, and globally, the incidence was also generally higher in males than in females up to the age of 69 (Supplementary Figures 3A, 5A). By 2021, although the peak incidence for males in China still occurred in the 0–5 age group, the peak for females had shifted to the 55–59 age group (Figure 6A). In the United States, the peak incidence for both males and females occurred in the 70–74 age group (Supplementary Figure 4A), while globally, the peak incidence for both males and females occurred in the 65–69 age group (Supplementary Figure 6A). Overall, males exhibited higher incidence rates than females in China, the United States, and Globally, particularly in those under the age of 69. In terms of prevalence, in 1990, the peak prevalence of MND in China for both males and females occurred in the 20–24 age group (Figure 5B), while in the United States, the peak prevalence occurred in the 65–69 age group (Supplementary Figure 3B), and Globally, the peak prevalence for males and females occurred in the 20–24 and 25–29 age groups, respectively (Supplementary Figure 5B). In China, except for the 35–39 age group, males had higher prevalence than females in all age groups under 54 (Figure 5B). In the United States, males had higher prevalence than females in all age groups under 74 (Supplementary Figure 3B), and Globally, males had higher prevalence than females in all age groups under 69 (Supplementary Figure 5B). By 2021, the peak prevalence in China for both males and females had shifted to the 30–34 age group (Figure 6B), while the United States and Global prevalence continued to peak at 70–74 years (Supplementary Figures 4B, 6B). In China, males had higher prevalence in all age groups under 34, but females had higher prevalence in groups over 34 (Figure 6B). In the United States and Globally, males had higher prevalence than females in all age groups under 79 (Supplementary Figures 4B, 6B). Regarding mortality, in 1990, the peak mortality for MND in China and globally occurred in the 0–5 age group (Figure 5C and Supplementary Figure 5C), while in the United States, the peak mortality for males occurred in the 65–69 age group and for females in the 70–74 age group (Supplementary Figure 3C). In China and Globally, except for the 45–49 age group, males had higher mortality than females in all age groups under 69 (Figure 5C and Supplementary Figure 5C), while in the United States, males had higher mortality than females in all age groups under 69, except for the 0–14 age group (Supplementary Figure 3C). By 2021, the peak mortality in China shifted to the 65–69 age group (Figure 6C), while in the United States and Globally, the peak mortality remained in the 70–74 age group (Supplementary Figures 4C, 6C). In China, males had higher mortality than females in all age groups under 79 (Figure 6C), and in the United States and Globally, males had higher mortality than females in most age groups under 79 (Supplementary Figures 4C, 6C). In terms of DALYs, in 1990, the peak DALYs for both males and females in China and globally occurred in the 0–5 age group (Figure 5D and Supplementary Figure 5D), while in the United States, the peak DALYs occurred in the 65–69 age group (Supplementary Figure 3D). In all age groups under 69, males had higher DALYs than females in China, the United States, and Globally (Figure 5D and Supplementary Figure 3D, 5D). By 2021, the peak DALYs for both males and females in China had shifted to the 55–59 age group (Figure 6D), while in the United States, the peak DALYs for males occurred in the 60–64 age group and for females in the 65–69 age group (Supplementary Figure 4D). Globally, both males and females had peak DALYs in the 65–69 age group (Supplementary Figure 6D). In China and Globally, males had higher DALYs than females in all age groups under 79 (Figure 6D and Supplementary Figure 6D), and in the United States, males had higher DALYs than females in all age groups under 79 except for the 10–14 age group (Supplementary Figure 4D).

#### Decomposition analysis of the factors influencing the incidence, prevalence, mortality, and DALYs of MND in China, the United States, and globally

**Figure 7:**
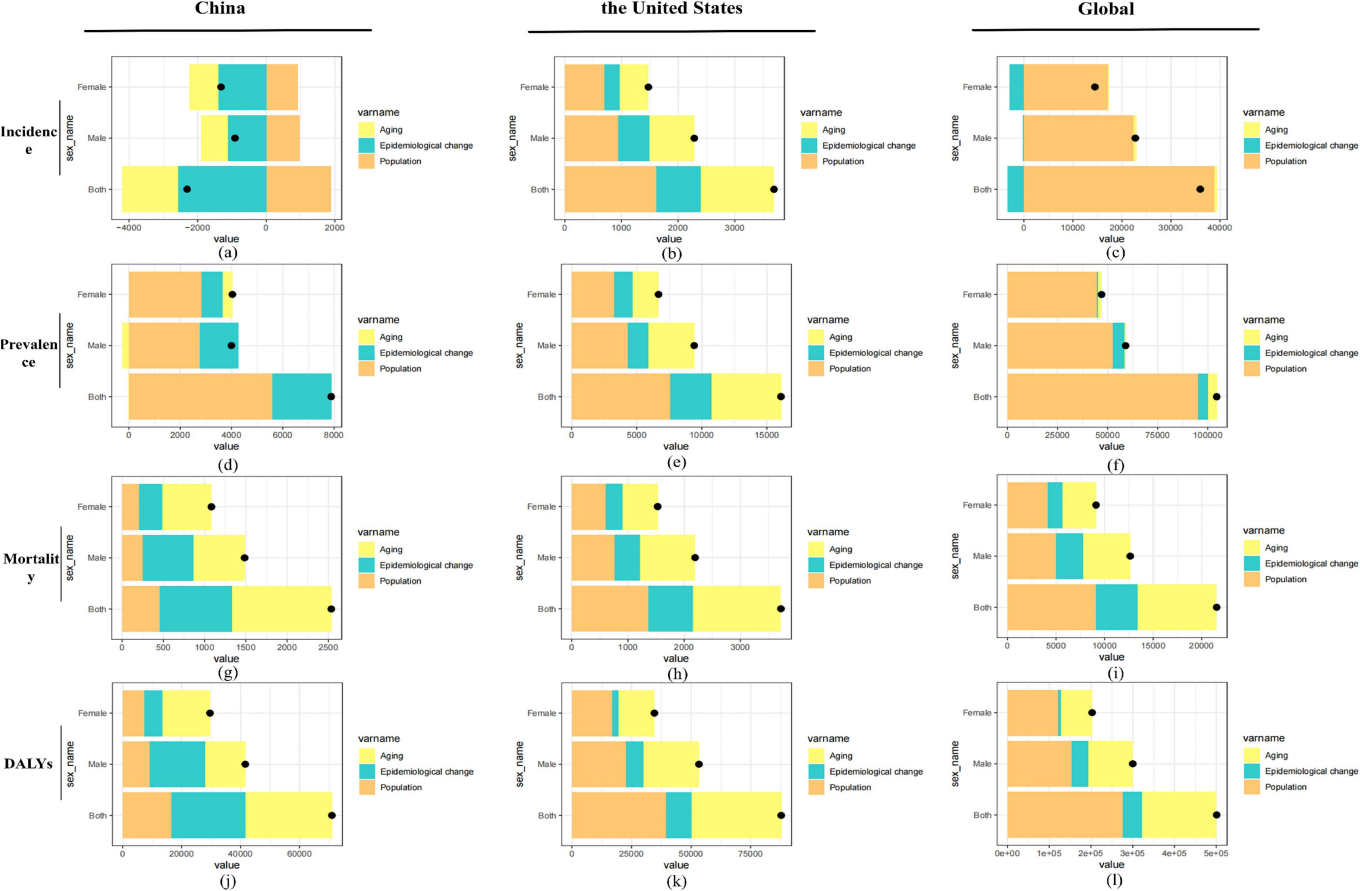
Decomposition analysis of factors influencing the incidence **(A–C)**, prevalence **(D–F)**, mortality **(G–I)**, and DALYs **(J–L)** of MND in China, the United States, and globally. Contributions of aging, epidemiological changes, and population growth are displayed for both sexes, males, and females, with black dots indicating the net effect of all factors combined.

This study performed a decomposition analysis of the factors influencing the incidence, prevalence, mortality, and DALYs of MND in China, the United States, and globally, revealing the differential impacts of aging, epidemiological changes, and population factors across regions and sexes. The results indicate that globally, population factors, particularly population aging, are the main drivers of the increase in MND indicators, especially in terms of incidence and prevalence. In contrast, aging and epidemiological changes have a smaller impact on Global MND incidence, highlighting the role of population aging in exacerbating the MND burden (Figures 7C,F,I,L,). In both China and the United States, MND incidence, mortality, and DALYs are primarily driven by aging, with aging having a particularly significant effect on mortality (Figures 7G,H), reflecting the increasing MND burden in the aging societies of these two countries. In China, aging and epidemiological changes have a suppressive effect on the incidence across all sex groups, with women being more affected, while population factors have driven an increase in incidence (Figure 7A). In the United States, aging, epidemiological changes, and population factors have collectively driven an increase in incidence across all sex groups, with men generally showing a higher rate of increase than women (Figure 7B). Globally, although aging and epidemiological changes have a smaller impact on incidence, population factors have significantly driven the global increase in MND incidence (Figure 7C). Gender differences exhibit a clear asymmetry in the various indicators, with men generally experiencing a higher increase in incidence, mortality, and DALYs than women, particularly on a Global scale. In China, aging has led to an increase in female prevalence, while male prevalence has decreased, and epidemiological changes and population factors have driven the overall increase in prevalence (Figure 7D). In the United States, men are more influenced by aging and population factors, while women’s prevalence is more affected by epidemiological changes (Figure 7E). Globally, population factors have a more significant effect on men, while epidemiological changes have a relatively smaller impact on the prevalence in both sexes (Figure 7F).

Overall, population factors, particularly aging, are the primary drivers of the increases in MND incidence, prevalence, mortality, and DALYs worldwide. In China and the United States, MND mortality and DALYs are mainly influenced by aging (Figures 7G,H,J,K). Globally, gender differences further amplify these effects. The varying impacts of epidemiological changes and population factors across regions and sexes suggest that these differences should be fully considered when formulating public health policies and interventions for MND.

#### Prediction analysis of ASIR, ASPR, ASMR, and ASDR for MND in China, the United States, and globally from 2022 to 2041

**Figure 8:**
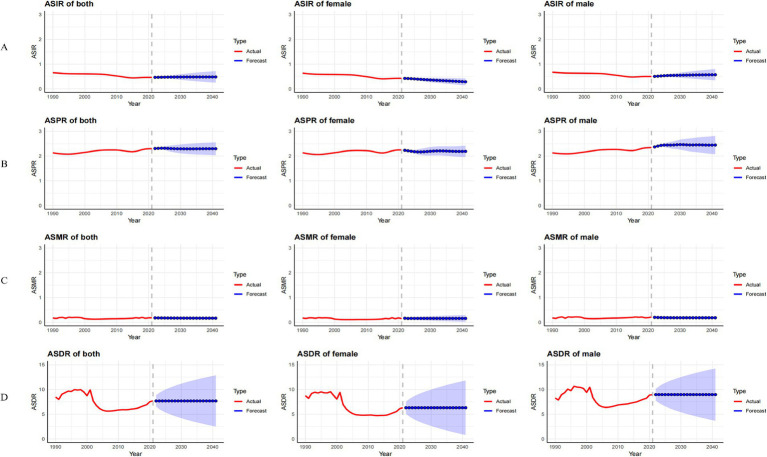
Observed trends (1990–2021) and ARIMA-based projections (2022–2041) of the age-standardized incidence rate (ASIR; **A**), prevalence rate (ASPR; **B**), mortality rate (ASMR; **C**), and disability-adjusted life years rate (ASDR; **D**) of MND in China. The red lines represent historical data, while the blue lines with shaded confidence intervals show forecasts.

**Figure 9:**
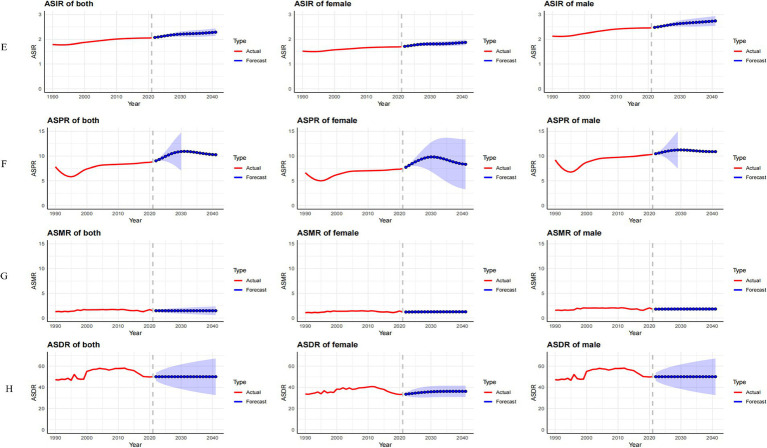
Observed trends (1990–2021) and ARIMA-based projections (2022–2041) of the age-standardized incidence rate (ASIR; **E**), prevalence rate (ASPR; **F**), mortality rate (ASMR; **G**), and disability-adjusted life years rate (ASDR; **H**) of MND in the United States. The red lines represent historical data, while the blue lines with shaded confidence intervals show forecasts.

**Figure 10:**
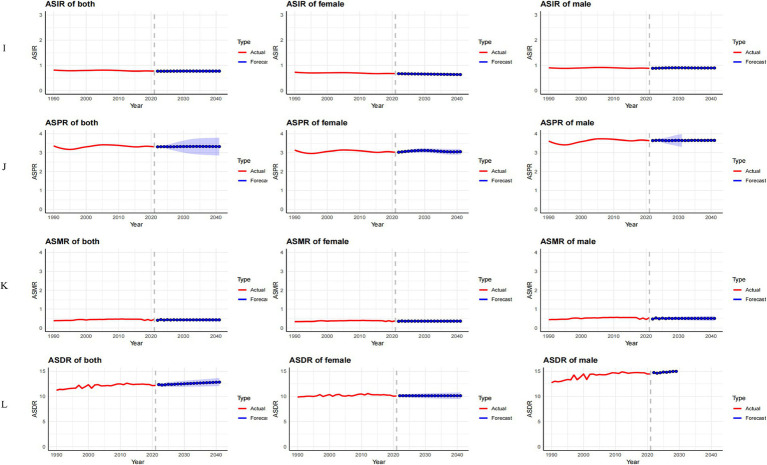
Observed trends (1990–2021) and ARIMA-based projections (2022–2041) of the age-standardized incidence rate (ASIR; **I**), prevalence rate (ASPR; **J**), mortality rate (ASMR; **K**), and disability-adjusted life years rate (ASDR; **L**) of MND in Global. The red lines represent historical data, while the blue lines with shaded confidence intervals show forecasts.

In China, between 2022 and 2041, the ASIR for male MND is predicted to gradually decline, while the ASIR for females is expected to increase (Figure 8A). Despite the gender differences, the overall incidence rate remains relatively stable, suggesting that the driving factors for MND incidence are largely consistent. The ASPR, ASMR, and ASDR are predicted to remain stable (Figures 8B–D), likely reflecting ongoing improvements in MND management in China, including advancements in disease diagnosis, treatment, and rehabilitation services. These medical interventions contribute to reducing mortality and disability burden. In the United States, the ASIR for MND is projected to rise from 2022 to 2041, with a more pronounced increase in males (Figure 9E). This trend may be associated with population aging and improved diagnosis rates. For ASPR, the trend shows more dynamic changes, particularly with a significant increase between 2022 and 2023, followed by a decline after 2023, especially among females (Figure 9F). This shift could be linked to increased health awareness and the widespread adoption of early intervention strategies among women. However, both the ASMR and ASDR are expected to remain stable (Figures 9G,H), likely due to advancements in supportive care and early intervention, particularly in high-income groups where medical resources are more abundant, effectively controlling mortality and disability burden. Globally, from 2022 to 2041, the ASIR, ASPR, and ASMR are expected to remain stable in both male and female populations (Figures 10I–K). This stability reflects the global trend of consistent MND incidence, which may be due to improved disease diagnosis, reporting standards, and the widespread adoption of disease management technologies. However, despite the stable incidence rates, the ASDR is projected to rise, with a more significant increase in females (Figure 10L). This upward trend may be attributed to the global aging population and the relatively limited healthcare resources in some low- and middle-income countries.

## Discussion

This study provides a comprehensive assessment of the changing burden and driving factors of MND in China, the United States, and globally from 1990 to 2021, with projections for 2022 to 2041.

In China, the ASIR of MND showed a declining trend despite an overall increase in case numbers. This decline is largely attributed to the effects of population aging (19, 20) and age standardization. Meanwhile, advancements in medical technology and the expansion of early screening have led to improved detection and reporting of early-stage cases (21, 22), contributing to the continued rise in both case numbers and the ASPR. In parallel, mortality and DALYs have increased significantly, indicating that while survival has improved, MND patients–particularly the elderly–continue to suffer from progressive functional deterioration. Although the ASDR showed a slight decline, likely reflecting delayed disease progression due to better interventions, the persistent nature of MND means the long-term burden of disability remains high. Additionally, the refinement of diagnostic and reporting systems has enabled more comprehensive case capture, further amplifying the apparent disease burden. In the United States, the incidence and prevalence of MND have steadily increased over the past three decades. This trend is closely associated with the rapid aging of the population, significant advancements in diagnostic technologies, and greater public awareness of neurodegenerative diseases. In elderly populations, the expansion of early screening and interventions has notably improved diagnostic rates. The implementation of the Affordable Care Act (23) has further enhanced access to healthcare and health insurance, facilitating earlier diagnosis and registration. Nevertheless, the continuing rise in mortality and DALYs indicates that, despite prolonged survival, MND patients face enduring functional decline and diminished quality of life. This is especially evident among the elderly, where extended life expectancy often coincides with severe disability, placing increasing pressure on long-term care systems and social support infrastructures. In short, while the U.S. has made progress in MND recognition and treatment, it still faces systemic challenges in chronic disease management and functional rehabilitation. Globally, although ASIR and ASPR have shown only slight declines due to standardization, the absolute number of incident and prevalent cases has increased significantly–largely driven by global aging, particularly in developed countries. The growth of the elderly population has intensified MND incidence, while age-standardization has masked the full extent of the increase. Meanwhile, the WHO’s Global Strategy and Action Plan on Ageing and Health (24) and Global Action Plan for the Prevention and Control of Noncommunicable Diseases (25) have improved global capacity for chronic disease management and elder care, leading to better identification and earlier intervention in MND cases. The parallel rise in mortality and DALYs further underscores the severe and disabling nature of MND, which, in the context of increased life expectancy, poses significant challenges for long-term care and rehabilitation. In high-income countries with relatively robust healthcare systems (26), extended survival is a positive development but also substantially increases the burden of years lived with disability. These trends highlight the need to not only strengthen healthcare systems globally, but also to address disparities in access and capacity, especially in low-resource settings, by building a more resilient and equitable global response to chronic neurological disorders.

Joinpoint regression analysis revealed that between 1990 and 2021, ASIR, ASPR, ASMR, and ASDR underwent significant stage-specific fluctuations with distinct regional patterns. In China, ASIR declined steadily before 2015, likely reflecting the impact of healthcare reforms, the expansion of primary care, and national chronic disease screening programs (5). However, ASIR began to rebound after 2015, indicating the counterbalancing effects of accelerated aging and improved disease awareness. ASPR exhibited a gradual upward trend, consistent with prolonged survival and improved chronic disease management. ASMR and ASDR showed marked variability–rising during early periods likely due to diagnostic delays and resource limitations, falling during phases of improved healthcare access, and rising again in recent years as the aging population has grown and long-term care resources have become increasingly strained. As an irreversible, progressive neurodegenerative condition (1), MND imposes a dual burden of mortality and disability, particularly among long-term survivors and elderly patients (27, 28). In the United States, ASIR increased consistently, especially after 1995, driven by aging, lifestyle factors, and the widespread availability of advanced diagnostics such as neuroimaging and genetic testing. The initial decline in ASPR during 1990–1995 may reflect inconsistent diagnostic criteria and underreporting, but the subsequent sharp rebound was likely due to health policy advances (29) and growing public health attention to neurodegenerative conditions (30, 31). ASMR and ASDR fluctuated over time–initial increases driven by aging and improved case recognition, periods of stabilization due to early interventions, and recent rises associated with growing numbers of elderly survivors and healthcare system pressures. At the global level, ASIR and ASPR experienced a three-phase pattern of decline, rise, and subsequent decline from 1990 to 2021, reflecting disparities in healthcare development and diagnostic capacity across countries. Meanwhile, ASMR and ASDR continued to rise, especially among older adults. This trend is driven by the combined effects of global aging, environmental pollution, lifestyle changes, and stark inequalities in healthcare resource distribution (4, 5). The rising global burden of MND highlights not only biomedical challenges, but also the need for coordinated health policy responses. This includes scaling up prevention strategies, building sustainable long-term care systems, and ensuring equitable access to neurodiagnostic technologies–particularly in low- and middle-income countries–within a globally resilient framework for noncommunicable disease control.

Decomposition analysis reveals that population factors, particularly population aging, are the main drivers behind the increased incidence, prevalence, mortality, and DALYs of MND globally. In both China and the United States, the incidence, mortality, and DALYs of MND are primarily driven by aging, with the impact of age being particularly significant on mortality rates. This reflects the increasing burden of MND on aging societies in both countries. In contrast, prevalence is mainly influenced by population factors. With the development of socio-economic and medical levels, the survival time of MND patients has increased, and the burden of MND in aging populations continues to rise. There is an urgent need to focus on healthcare infrastructure, including the development of integrated care models that incorporate long-term care, rehabilitation, and palliative care for elderly patients.

Forecast analysis indicates significant differences in the projected trends of MND burden over the next 20 years (2022–2041) in China, the United States, and globally. In China, the ASIR is expected to decrease in males and increase in females, while the ASPR is predicted to remain stable. Both ASMR and ASDR are anticipated to stay stable. In the United States, the ASIR is expected to continue rising, especially among males, while the ASPR will significantly increase between 2022 and 2030, followed by a decline. ASMR and ASDR are projected to remain stable. Globally, the ASIR, ASPR, and ASMR are expected to stabilize, but the ASDR for females is anticipated to rise. Given the complex etiology and heterogeneity of MND, there is uncertainty in the prediction results. Future research should focus on early diagnosis, treatment advancements, and the impact of social factors on MND burden trends.

This study highlights the gender and regional disparities in the burden of MND. Men have higher incidence, prevalence, and mortality rates, which are associated with genetic susceptibility, hormonal influences, and lifestyle factors (27). Although men bear a heavier burden, the burden of MND in elderly women has been increasing year by year, reflected by rising prevalence and DALYs. Therefore, gender-sensitive medical interventions are necessary, such as early detection, tailored healthcare services, and public awareness campaigns to improve recognition of MND in elderly women. Additionally, high-income countries benefit from strong healthcare systems that provide early diagnosis and intervention, thereby extending survival, reflecting advances in medical care. However, low and middle-income countries face poorer MND outcomes and higher mortality rates due to limited medical resources, diagnostic delays, and restricted treatment options (26). In China, the urban–rural gap has exacerbated the MND burden, with urban areas benefiting from improved healthcare facilities, while rural areas still face insufficient healthcare resources (32). Therefore, healthcare policies should focus on improving healthcare access in rural and underserved areas to ensure timely diagnosis and treatment for everyone.

## Study strengths and limitations

This study is based on the GBD 2021 database, which offers relatively high-quality data, and applies rigorous analytical approaches including Joinpoint regression, decomposition analysis, and ARIMA forecasting models–allowing for robust temporal and spatial comparisons. However, several limitations should be acknowledged. First, GBD data are influenced by national reporting systems and may underestimate the true burden of MND in countries with weak health surveillance. Second, the modeling relies on a series of assumptions (e.g., stationarity in ARIMA, linearity in Joinpoint regression), which may not fully capture the complexity of disease epidemiology. Third, changes in diagnostic criteria over time, underreporting, and improved disease recognition could affect trend interpretation. Lastly, the absence of socio-economic stratification limits our ability to explore health inequalities within countries.

## Future research directions

Future research should focus on improving data completeness and diagnostic accuracy, particularly in low- and middle-income countries. Advancing population-based prospective cohort studies and building patient-level databases are essential for tracking disease progression and evaluating intervention effectiveness. It is also recommended to integrate MND surveillance into national health information systems, strengthen access to neurodiagnostic tools, and improve long-term care systems. Additionally, further investigation is needed into the roles of environmental and occupational exposures, genetic susceptibility, and sex differences in the etiology and outcomes of MND.

## Conclusion

This study reveals that the incidence, prevalence, mortality, and DALYs of MND in China, the United States, and globally have significantly increased, driven mainly by population aging, advances in diagnostic technologies, and greater disease awareness. Despite healthcare improvements, MND-related mortality and long-term disability burdens, especially in the elderly, continue to rise. Gender and regional disparities are notable, with men bearing a higher MND burden, though the burden on elderly women has increased in recent years. Low- and middle-income countries, with limited medical resources, diagnostic delays, and fewer treatment options, face poorer outcomes and higher mortality rates. The study underscores the need for gender-sensitive medical interventions, early diagnostic technology promotion, and enhanced healthcare infrastructure, particularly in rural and underserved areas. It also emphasizes the complex etiology of MND, with population, socio-economic, and healthcare system factors influencing trends. Future research should focus on early diagnosis, treatment optimization, and the role of socio-economic factors. Ultimately, this study provides crucial data to inform global public health strategies and healthcare policies aimed at reducing the MND burden through targeted interventions.

## Data Availability

The datasets presented in this study can be found in online repositories. The names of the repository/repositories and accession number(s) can be found at: data for this study are accessible via the Institute for Health Metrics and Evaluation (IHME)’s online platform, found at https://vizhub.healthdata.org/gbd-results/.
