# Supplementary material for: Comparative analysis of trends in the burden of motor neuron disease in China, the United States, and globally from 1990 to 2021: projections for 2022–2041
Source: Front Neurol. 2025 May 16;16:1539889. doi: 10.3389/fneur.2025.1539889 (PMC12122310; doi:10.3389/fneur.2025.1539889)
Supplement: Supplementary file 1 [file Table_1.docx]

| Location | Sex name | Measure | 1990 | | 2021 | | 1990-2021 |
| --- | --- | --- | --- | --- | --- | --- | --- |
|  |  |  | All-ages cases | Age-standardized rates per 100,000 people | All-ages cases | Age-standardized rates per 100,000 people | AAPC |
|  |  |  | n(95%CI) | n (95% CI) | n(95%CI) | n (95% CI) | n (95% CI) |
| China | Both | Deaths | 1528(845,1953) | 0.15(0.09,0.19) | 3450(2220,4790) | 0.18(0.11,0.25) | 0.57*(0.26,0.88) |
|  |  | DAL Ys | 87564(50251,111668) | 7.99(4.67,10.15) | 122661(81013,167332) | 7.67(4.88,10.06) | -0.15(-0.51,0.22) |
|  |  | Prevalence | 25691(20344,31960) | 2.13(1.72,2.60) | 33342(27028,40366) | 2.30(1.84,2.80) | 0.26*(0.21,0.30) |
|  |  | Incidence | 6854(5928,7956) | 0.65(0.57,0.75) | 7324(5993,8709) | 0.46(0.39,0.54) | -1.10*(-1.22,-0.99) |
|  | Male | Deaths | 802(86,1189) | 0.15(0.02,0.23) | 1939(667,3131) | 0.21(0.07,0.33) | 0.95*(0.56,1.34) |
|  |  | DALYs | 45779(7256,66689) | 7.87(1.22,11.46) | 70894(27361,113107) | 8.94(3.44,13.64) | 0.38(-0.01,0.77) |
|  |  | Prevalence | 13281(10533,16576) | 2.13(1.72,2.62) | 16980(13774,20513) | 2.35(1.86,2.83) | 0.32*(0.28,0.36) |
|  |  | Incidence | 3667(3165,4241) | 0.67(0.58,0.78) | 3932(3269,4636) | 0.50(0.43,0.57) | -0.95*(-0.87,-1.02) |
|  | Female | Deaths | 726(609,841) | 0.15(0.13,0.17) | 1511(1187,1912) | 0.15(0.12,0.19) | 0.04(-0.37,0.44) |
|  |  | DALYs | 41785(36355,48767) | 8.20(7.13,9.58) | 51767(41917,64348) | 6.30(5.13,7.67) | -0.85*(-1.24,-0.46) |
|  |  | Prevalence | 12410(9852,15429) | 2.13(1.72,2.61) | 16362(13112,19981) | 2.25(1.79,2.75) | 0.18*(0.13,0.24) |
|  |  | Incidence | 3187(2758,3675) | 0.63(0.55,0.72) | 3392(2769,4072) | 0.42(0.35,0.50) | -1.27*(-1.42,-1.13) |
| the United States | Both | Deaths | 3890(3687,4008) | 1.26(1.20,1.29) | 8465(7800,8899) | 1.49(1.38,1.56) | 0.51*(0.12,0.91) |
|  |  | DALYs | 111652(107616,114636) | 39.71(38.48,40.72) | 210559(199250,218997) | 41.36(39.47,42.94) | 0.14(-0.22,0.50) |
|  |  | Prevalence | 22197(19257,25430) | 7.82(6.82,8.92) | 40495(37645,43576) | 8.82(8.26,9.51) | 0.39*(0.29,0.48) |
|  |  | Incidence | 5281(5004,5604) | 1.79(1.69,1.91) | 11089(10634,11559) | 2.06(1.98,2.14) | 0.45*(0.42,0.48) |
|  | Male | Deaths | 2035(1963,2087) | 1.52(1.46,1.56) | 4705(4441,4912) | 1.80(1.71,1.88) | 0.39*(0.07,0.72) |
|  |  | DALYs | 60845(59152,62402) | 46.99(45.72,48.22) | 120100(114053,125007) | 50.01(47.66,51.99) | 0.09(-0.15,0.34) |
|  |  | Prevalence | 12069(10460,13881) | 9.21(8.01,10.55) | 22755(21263,24480) | 10.33(9.74,11.13) | 0.37*(0.29,0.45) |
|  |  | Incidence | 2804(2653,2980) | 2.13(2.01,2.26) | 6154(5915,6410) | 2.46(2.37,2.57) | 0.48*(0.44,0.51) |
|  | Female | Deaths | 1855(1724,1928) | 1.06(1.00,1.10) | 3760(3366,4020) | 1.21(1.10,1.29) | 0.44(-0.10,0.99) |
|  |  | DALYs | 50807(48449,52383) | 33.49(32.28,34.43) | 90459(83918,95384) | 33.45(31.52,34.99) | -0.01(-0.43,0.42) |
|  |  | Prevalence | 10128(8779,11581) | 6.62(5.74,7.55) | 17740(16414,19155) | 7.46(6.96,8.05) | 0.37*(0.24,0.51) |
|  |  | Incidence | 2477(2339,2627) | 1.52(1.43,1.62) | 4935(4719,5152) | 1.70(1.63,1.78) | 0.36*(0.33,0.40) |
| Global | Both | Deaths | 15260 (14367,16043) | 0.38(0.36,0.40) | 39082(35757,42433) | 0.46(0.42,0.49) | 0.58*(0.45,0.70) |
|  |  | DALYs | 506146(462035-545050) | 11.22(10.39,11.98) | 1040566(963064,1123956) | 12.17(11.24,13.15) | 0.25*(0.09,0.42) |
|  |  | Prevalence | 161926(137006,189263 ) | 3.36(2.87,3.92) | 272732(236194,313676) | 3.31(2.86,3.80) | -0.04(-0.15,0.06) |
|  |  | Incidence | 36769(33068,41300) | 0.81(0.72,0.90) | 64178(58506,70270) | 0.77(0.70,0.84) | -0.15*(-0.18,-0.12) |
|  | Male | Deaths | 8012(7137,8579 ) | 0.44(0.40,0.46) | 21703(19623,23371) | 0.55(0.50,0.59) | 0.72*(0.54,0.91) |
|  |  | DALYs | 275249(230289,305186) | 12.73(11.00,13.82) | 592049(529600,642811) | 14.45(12.93,15.76) | 0.41*(0.19,0.62) |
|  |  | Prevalence | 84544(98825,71637) | 3.61(3.11,4.21) | 145479(126364,166787) | 3.64(3.16,4.16) | 0.04(-0.07,0.15) |
|  |  | Incidence | 19685(17693,22183) | 0.90(0.81,1.00) | 35138(32151,38498) | 0.88(0.81,0.96) | -0.06*(-0.09,-0.03) |
|  | Female | Deaths | 7248(6819,7577) | 0.33(0.31,0.35) | 17379(15419,19638) | 0.38(0.34,0.43) | 0.38*(0.21,0.55) |
|  |  | DALYs | 230897(219091,243419) | 9.86(9.38,10.36) | 448517(413228,496721) | 10.08(9.33,11.14) | 0.06(-0.13,0.25) |
|  |  | Prevalence | 77382(65287,90853) | 3.13(2.66,3.66) | 127253(109471,146510) | 3.02(2.60,3.52) | -0.12*(-0.21,-0.02) |
|  |  | Incidence | 17084(15300,19087) | 0.72(0.65,0.81) | 29040(26390,31866) | 0.67(0.61,0.74) | -0.25*(-0.29,-0.21) |
