# Supplementary material for: Comparative analysis of trends in the burden of motor neuron disease in China, the United States, and globally from 1990 to 2021: projections for 2022–2041
Source: Front Neurol. 2025 May 16;16:1539889. doi: 10.3389/fneur.2025.1539889 (PMC12122310; doi:10.3389/fneur.2025.1539889)
Supplement: Supplementary file 2 [file Supplementary_file_1.docx]

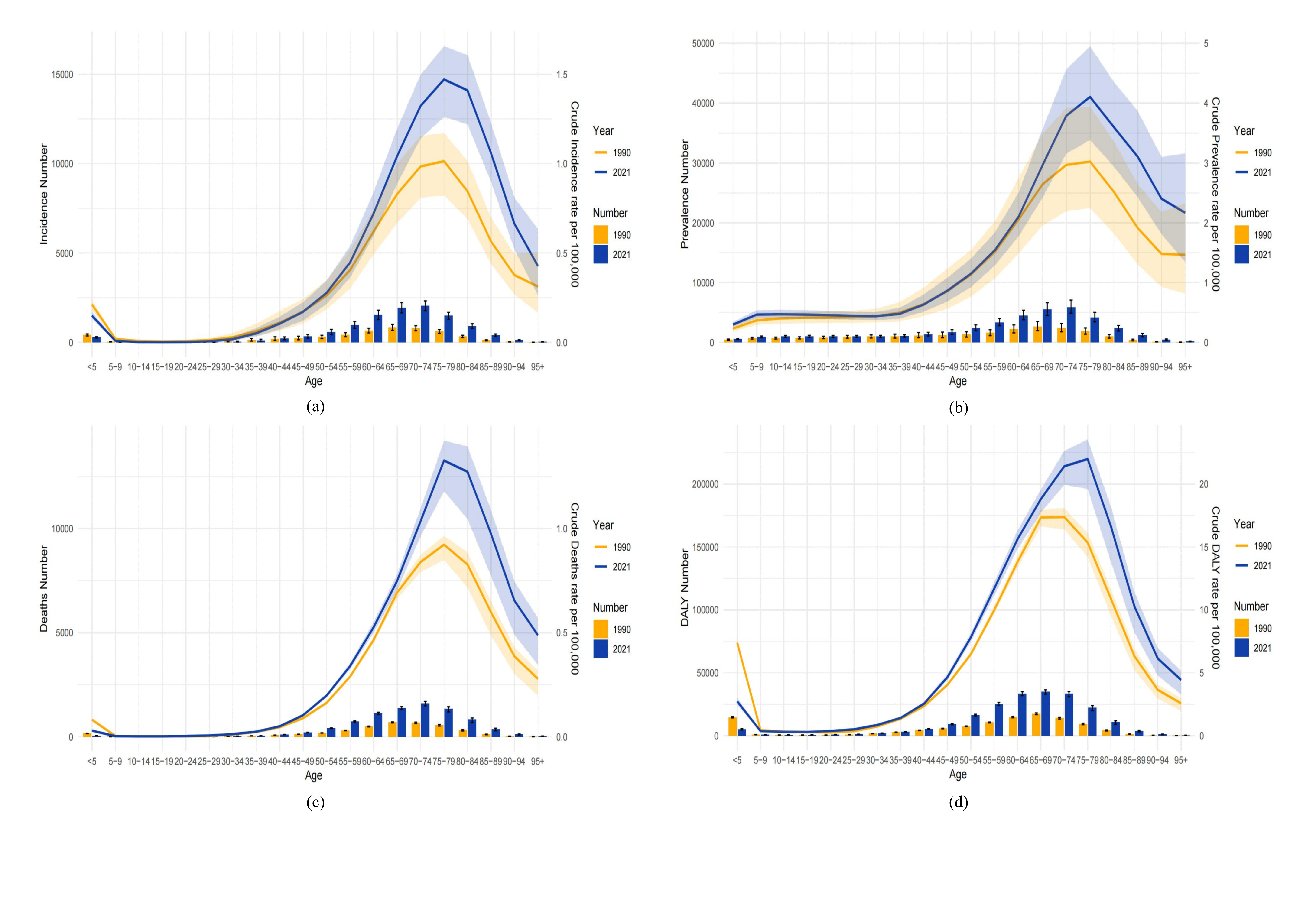


**Supplementary Figure 1** Comparative of the incidence, prevalence, deaths, and DALYs counts, along with their crude rates, by age group in the United States from 1990 and 2021. **(a)** Incident cases and CIR; **(b)** Prevalent cases and CPR; **(c)** Death cases and CMR; **(d)** DALYs counts and CDR; Bar charts represent counts; lines represent crude rates


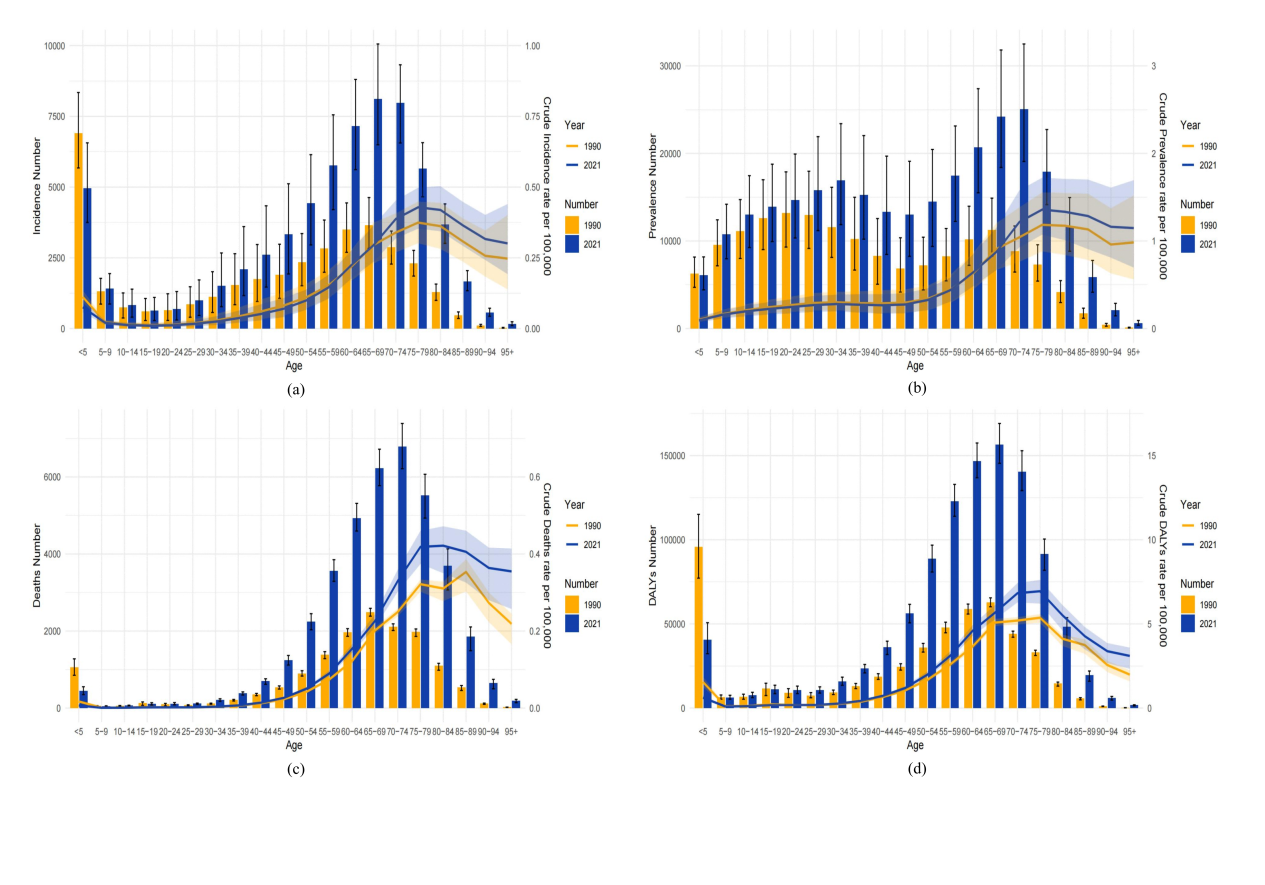


**Supplementary Figure 2** Comparative of the incidence, prevalence, deaths, and DALYs counts, along with their crude rates, by age group in Global from 1990 and 2021. **(a)** Incident cases and CIR; **(b)** Prevalent cases and CPR; **(c)** Death cases and CMR; **(d)** DALYs counts and CDR; Bar charts represent counts; lines represent crude rates


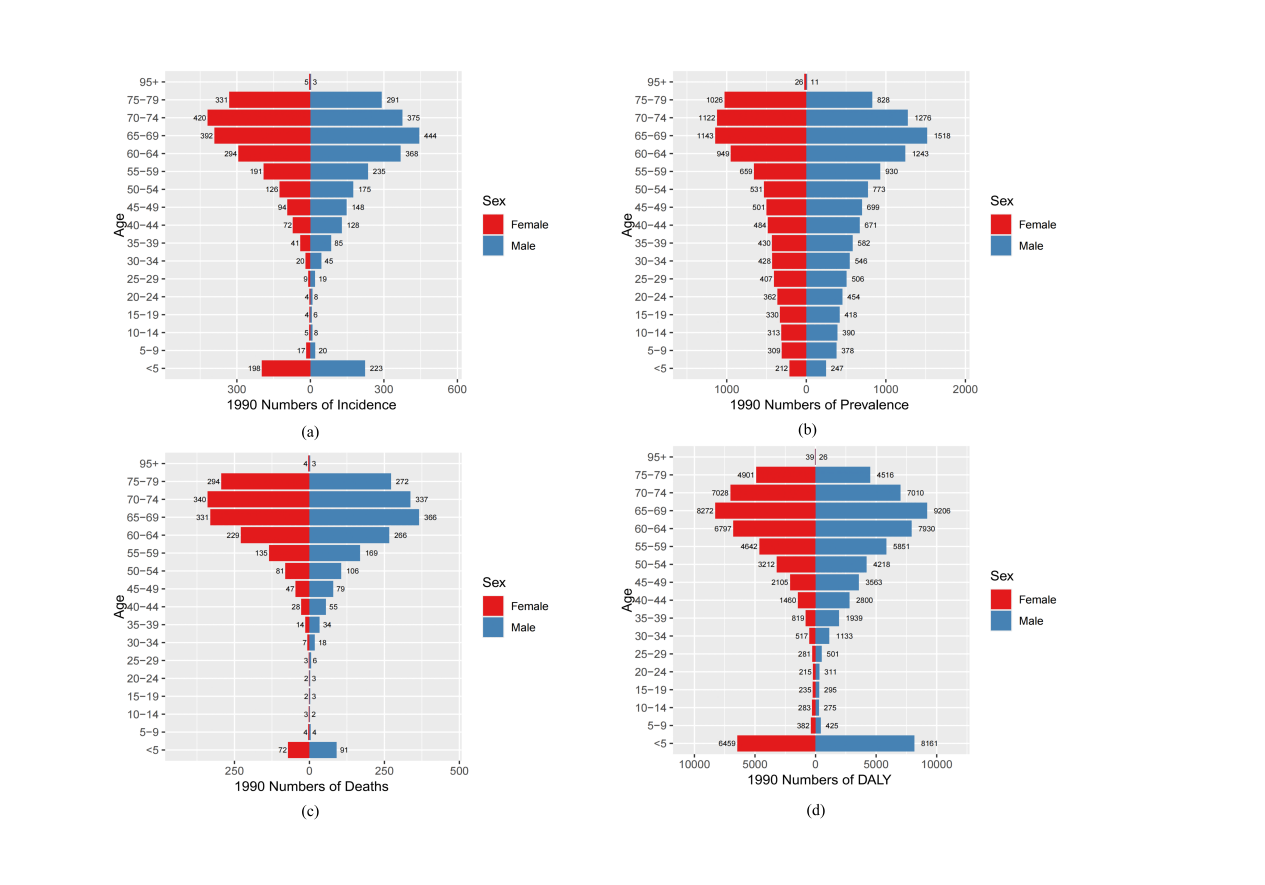


**Supplementary Figure 3** Comparison of the number of incidence, prevalence, mortality, and DALYs of MND in males and females of different age groups in the United States in 1990. **(a)** Incidence; **(b)** Prevalence; **(c)** Mortality; **(d)** DALYs


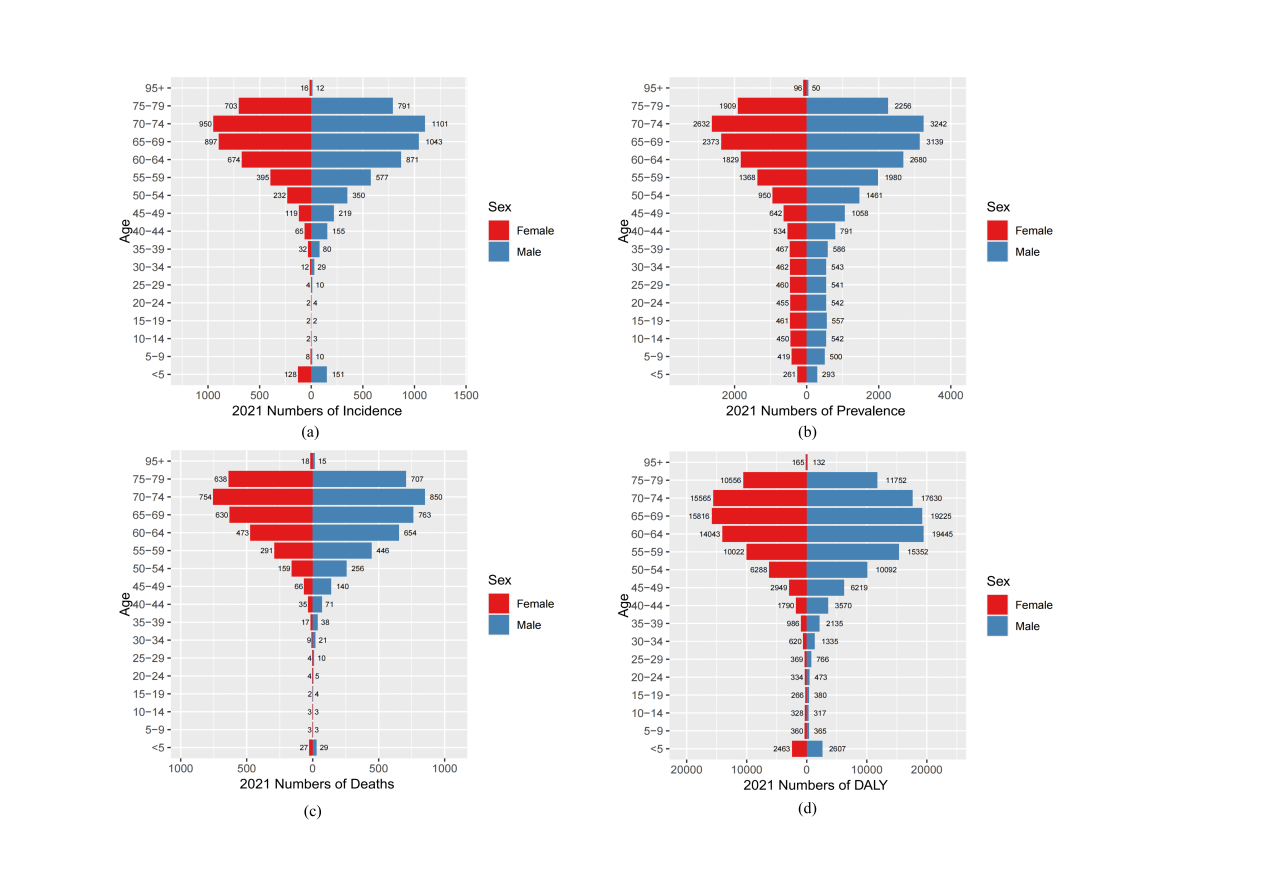


**Supplementary Figure 4** Comparison of the number of incidence, prevalence, mortality, and DALYs of MND in males and females of different age groups in the United States in 2021. **(a)** Incidence; **(b)** Prevalence; **(c)** Mortality; **(d)** DALYs


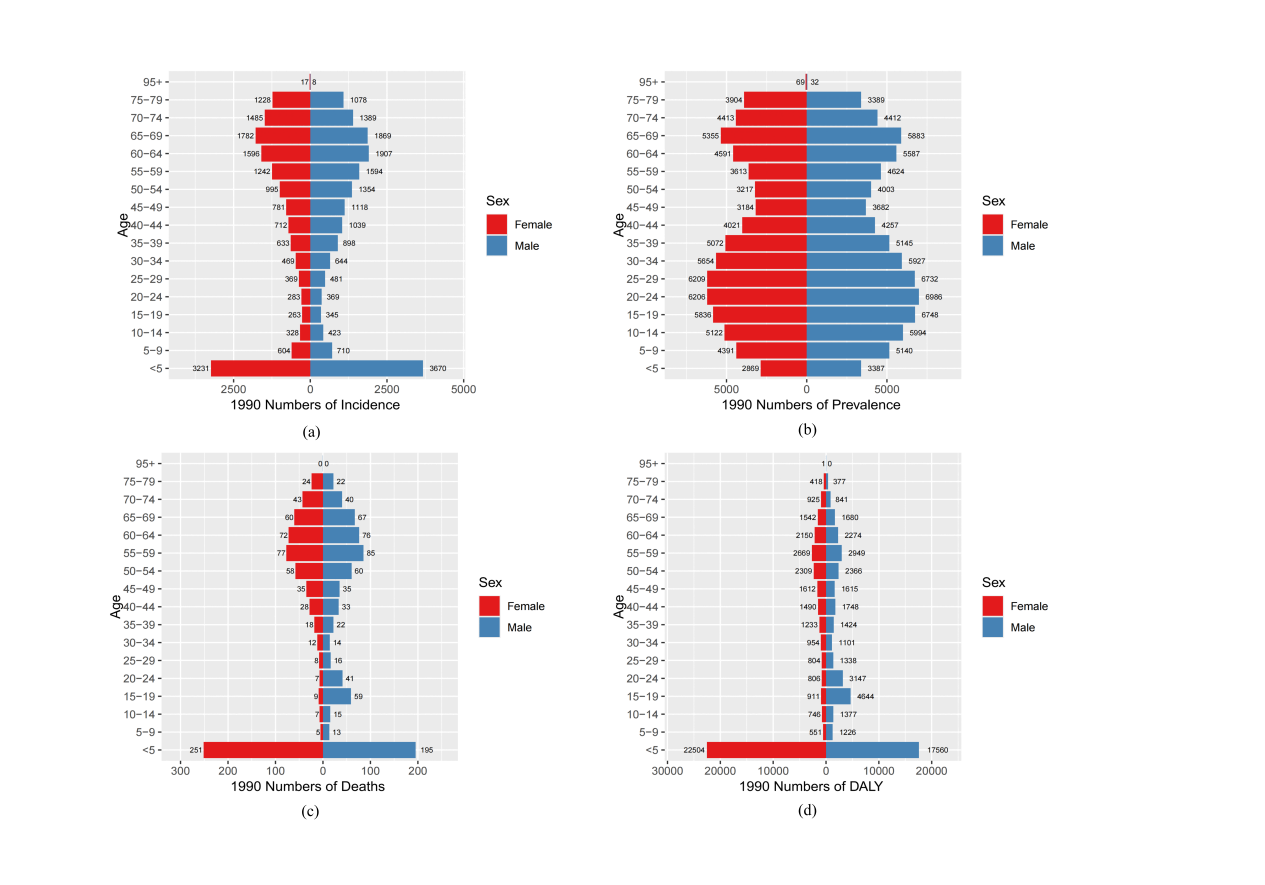


**Supplementary Figure 5** Comparison of the number of incidence, prevalence, mortality, and DALYs of MND in males and females of different age groups in Global in 1990. **(a)** Incidence; **(b)** Prevalence; **(c)** Mortality; **(d)** DALYs


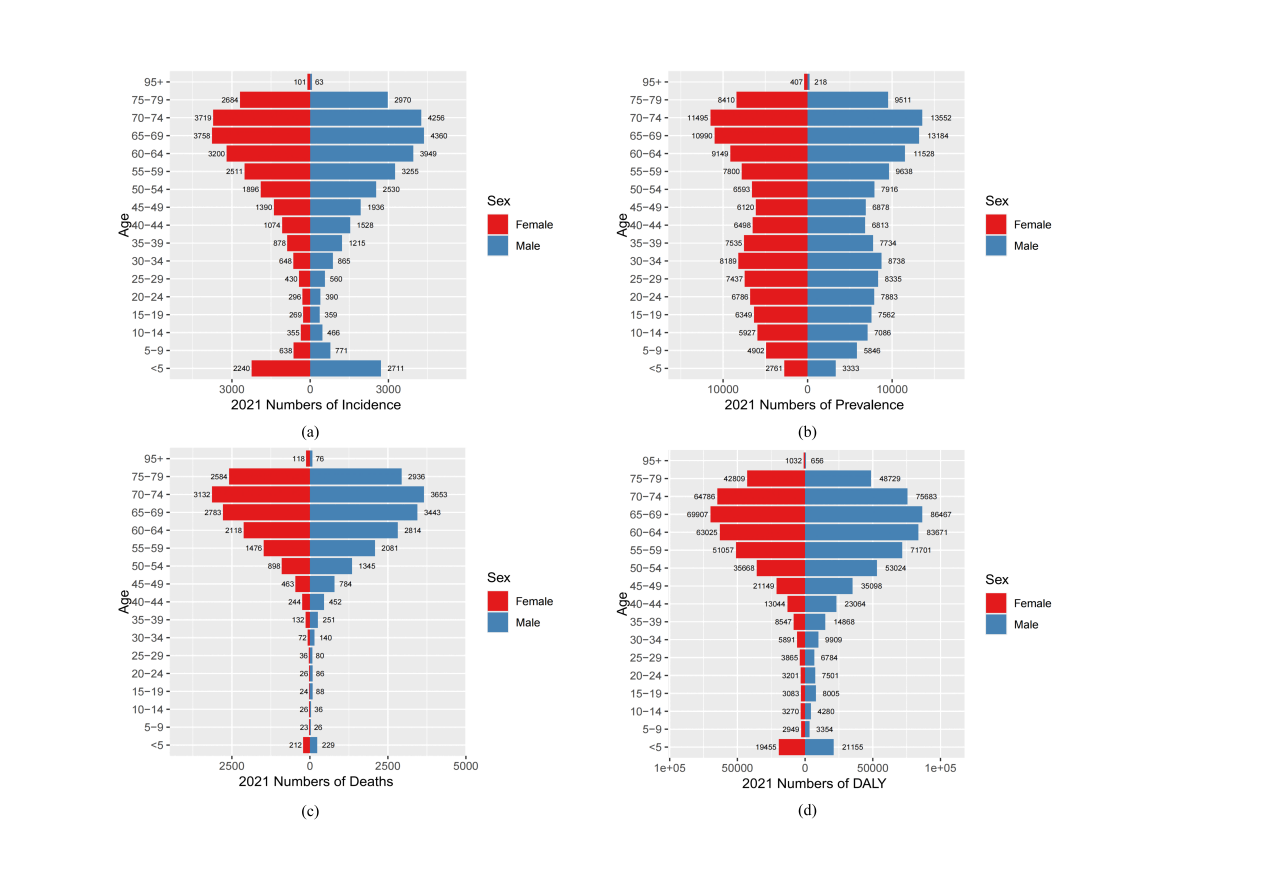


**Supplementary Figure 6** Comparison of the number of incidence, prevalence, mortality, and DALYs of MND in males and females of different age groups in Global in 2021. **(a)** Incidence; **(b)** Prevalence; **(c)** Mortality; **(d)** DALYs
