# Supplementary material for: Comparative analysis of trends in the burden of motor neuron disease in China, the United States, and globally from 1990 to 2021: projections for 2022–2041
Source: Front Neurol. 2025 May 16;16:1539889. doi: 10.3389/fneur.2025.1539889 (PMC12122310; doi:10.3389/fneur.2025.1539889)
Supplement: Supplementary file 3 [file Presentation_1.pptx]

## Slide 1
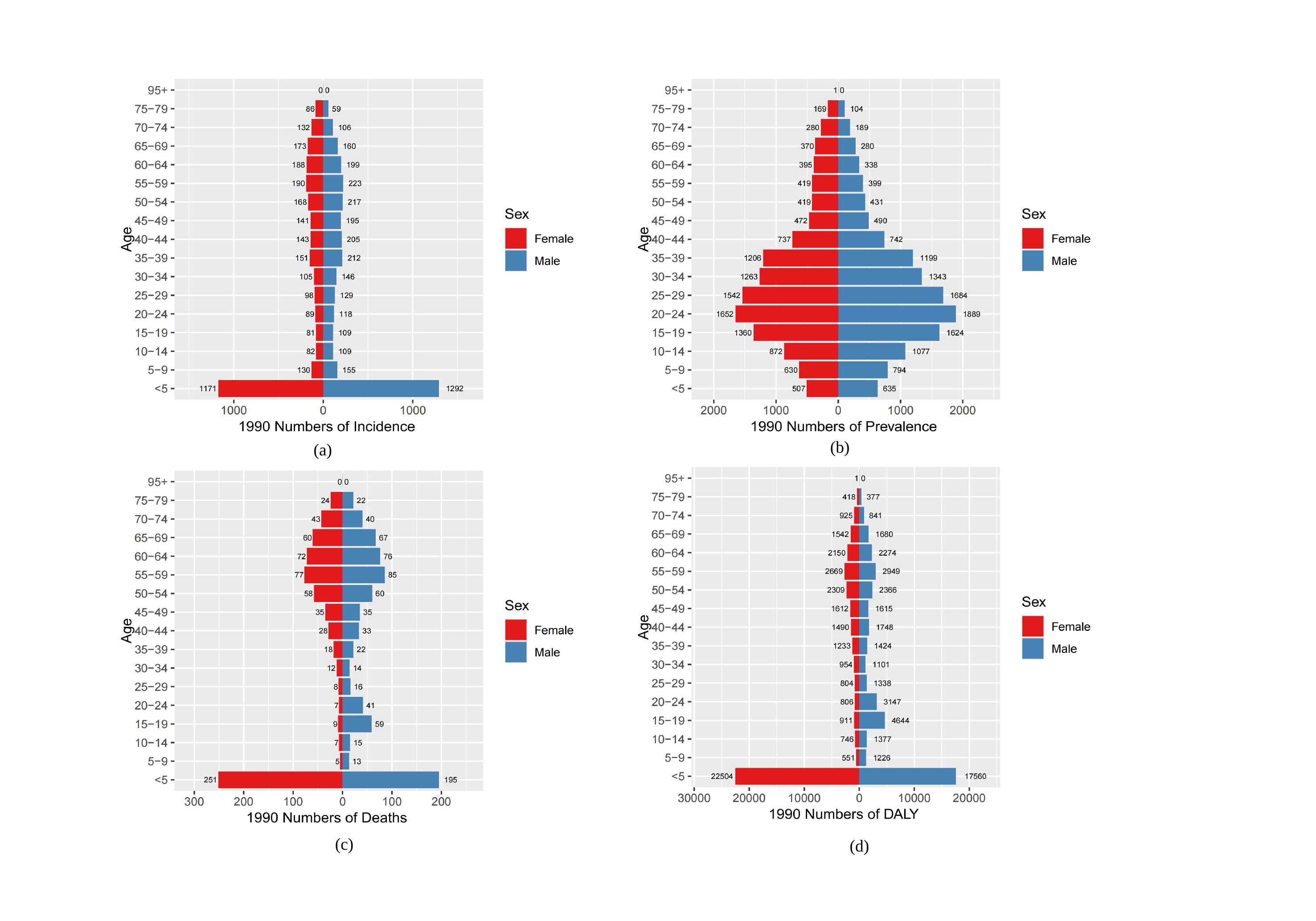

(b)
(a)
(c)
(d)

## Slide 2
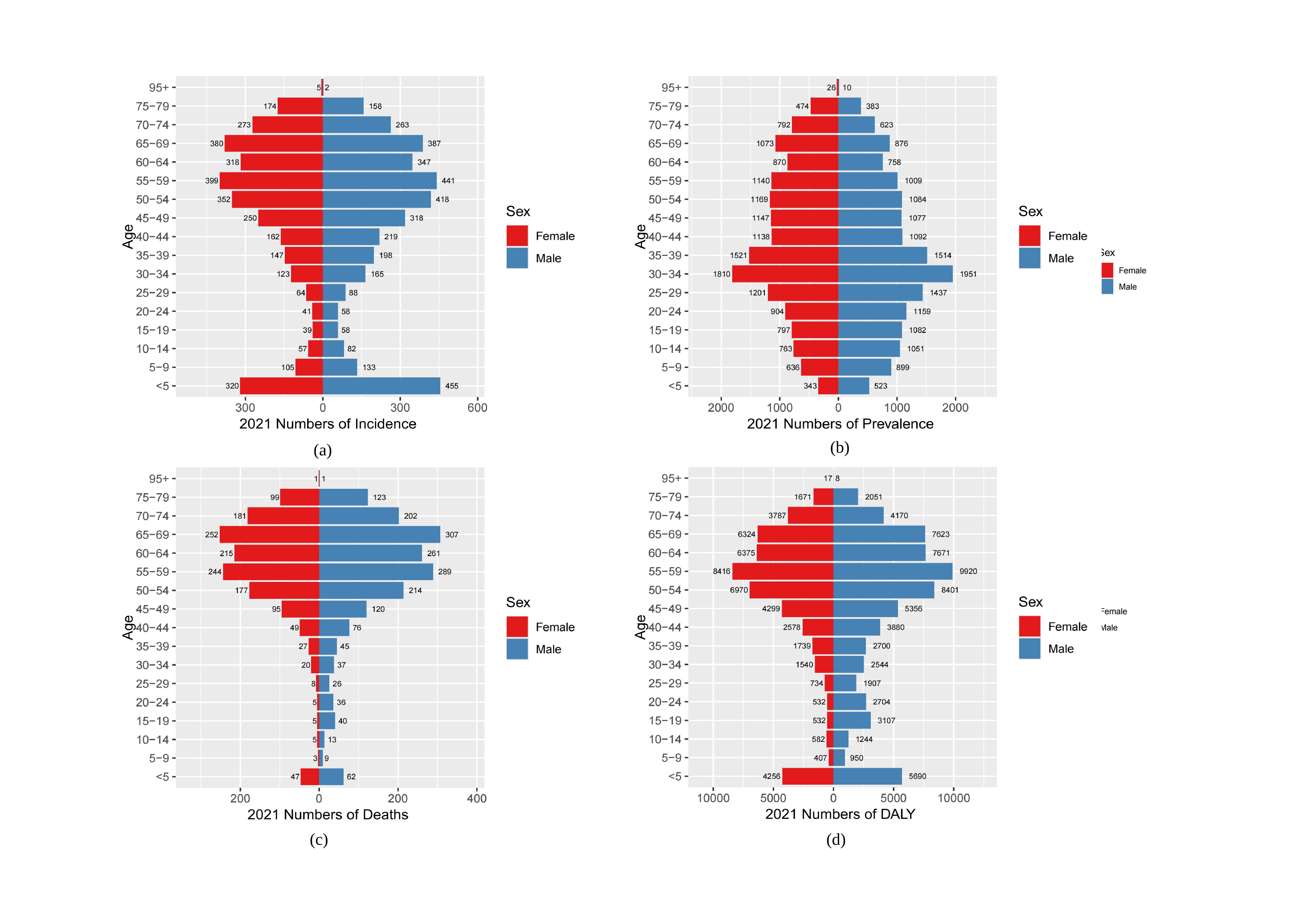

(b)
(a)
(c)
(d)

## Slide 3
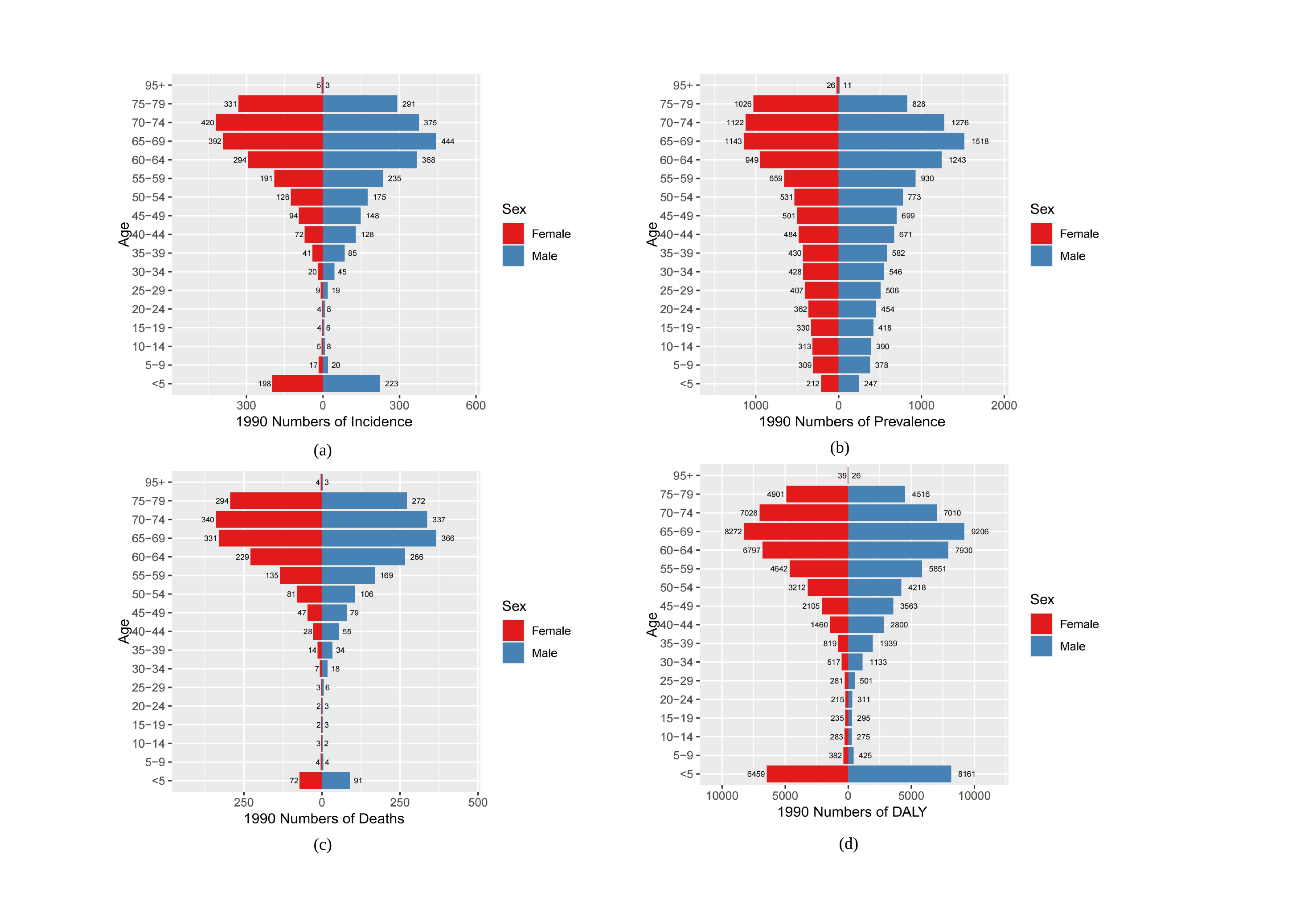

(b)
(a)
(d)
(c)

## Slide 4
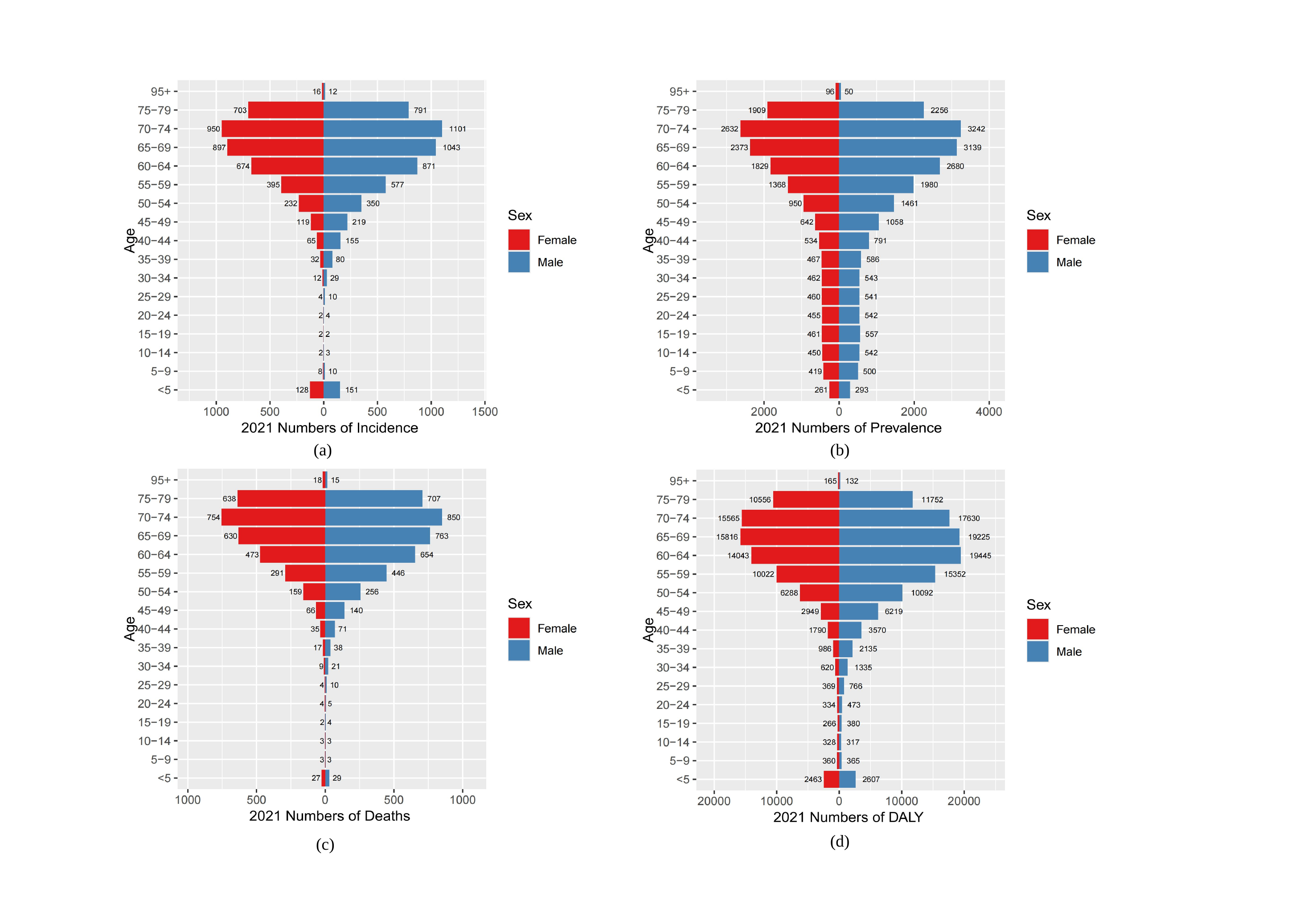

(b)
(a)
(d)
(c)

## Slide 5
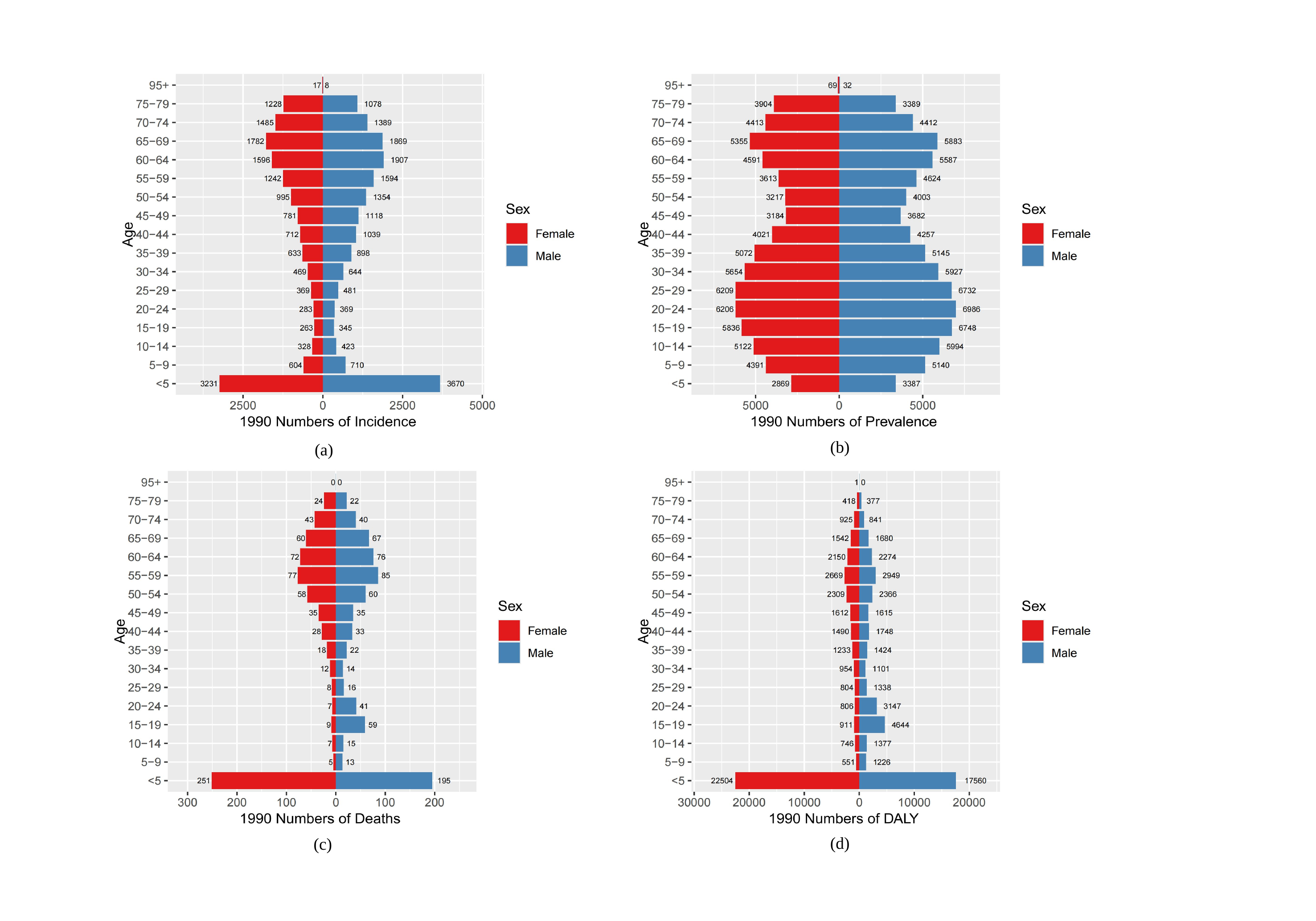

(b)
(a)
(d)
(c)

## Slide 6
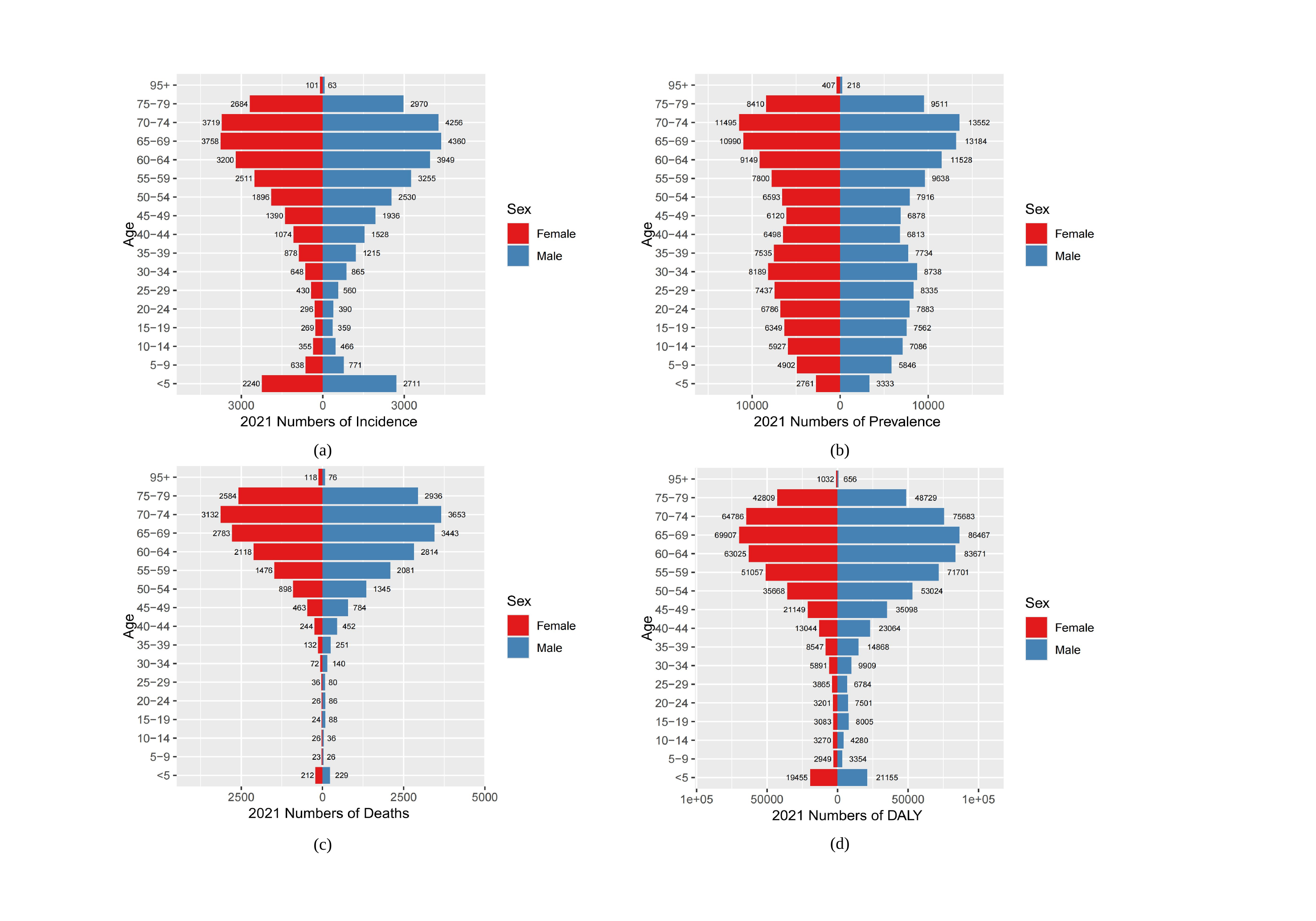

(a)
(b)
(d)
(c)

## Slide 7
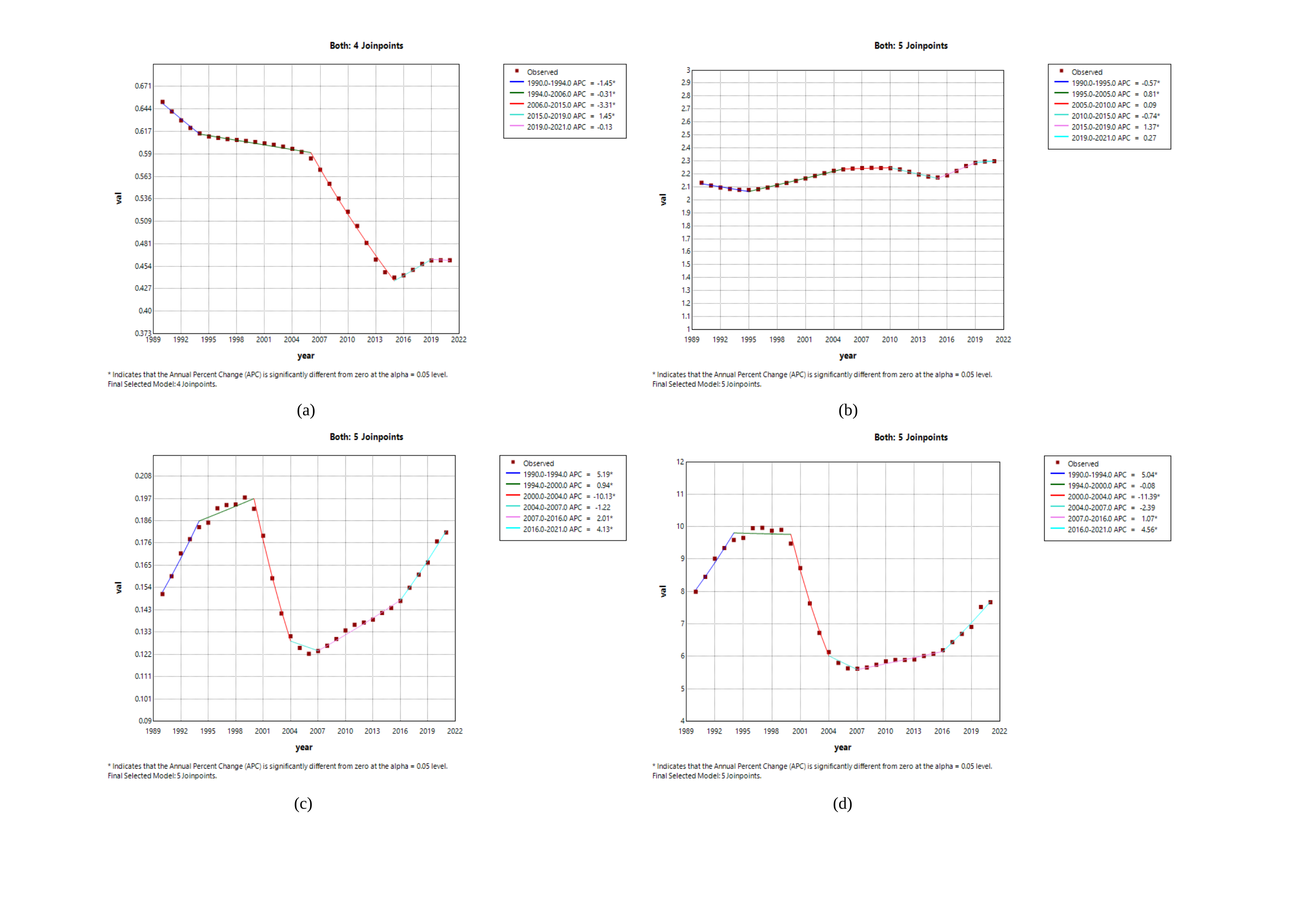

(a)
(b)
(c)
(d)

## Slide 8
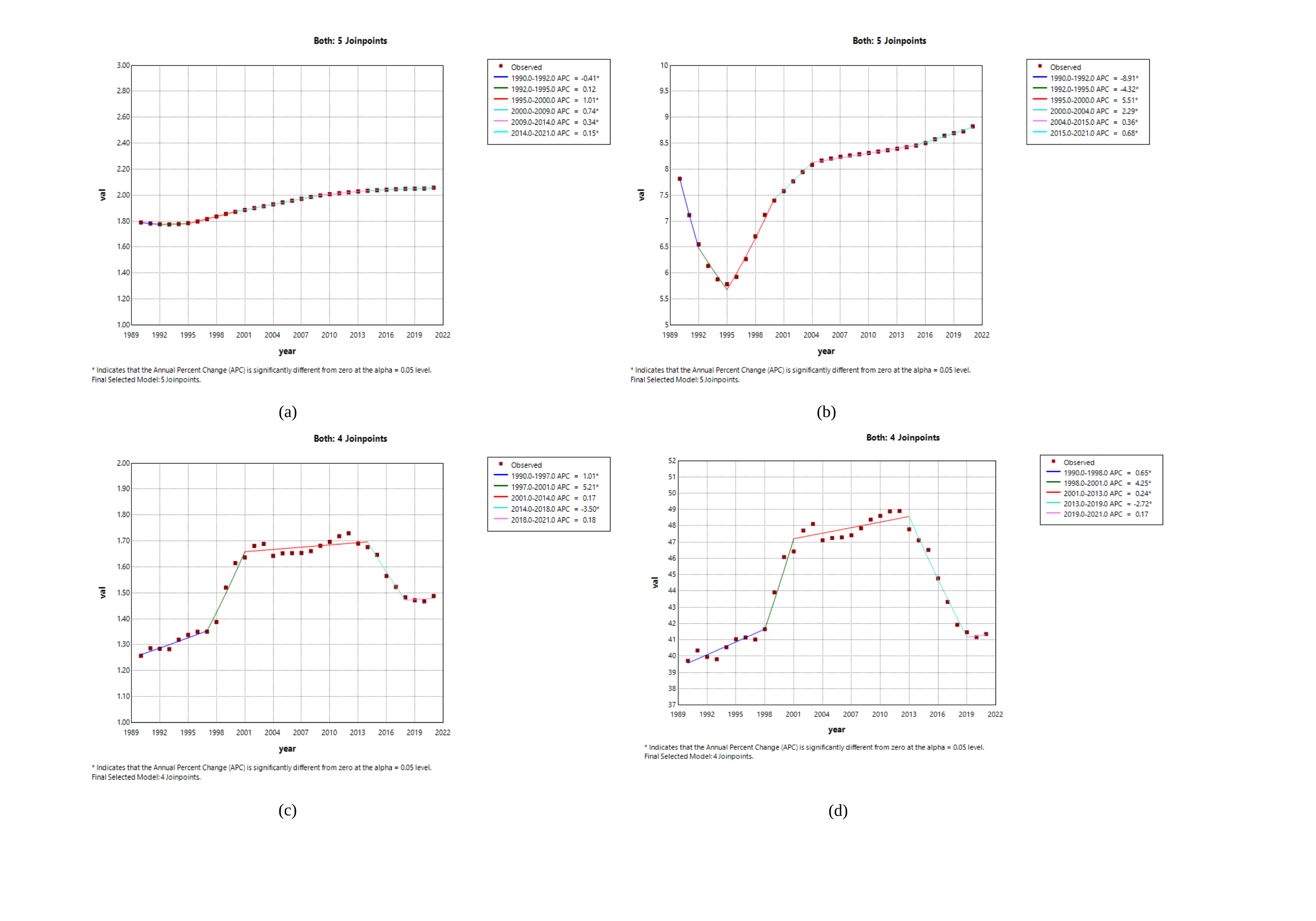

(a)
(b)
(c)
(d)

## Slide 9
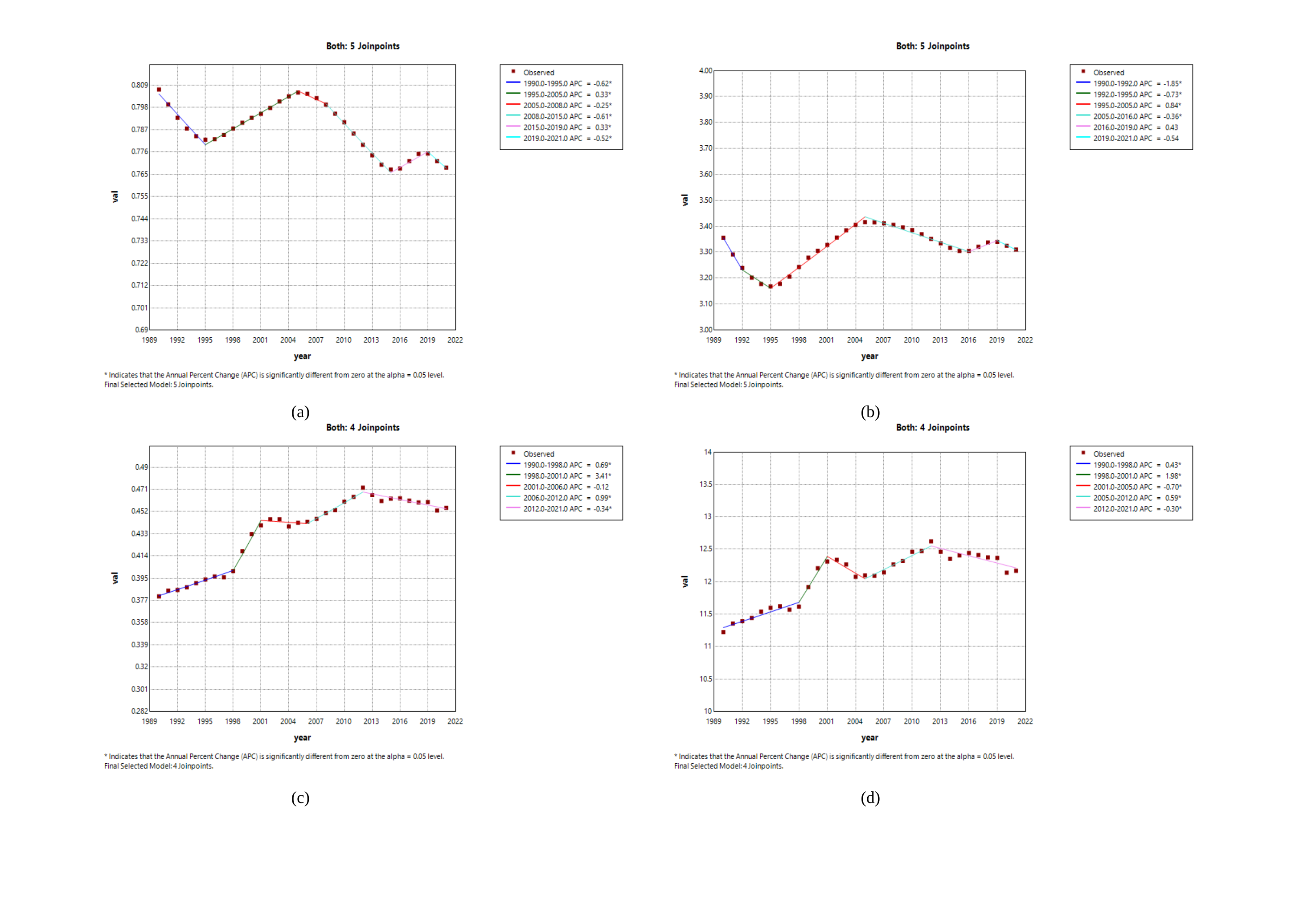

(a)
(b)
(c)
(d)

## Slide 10
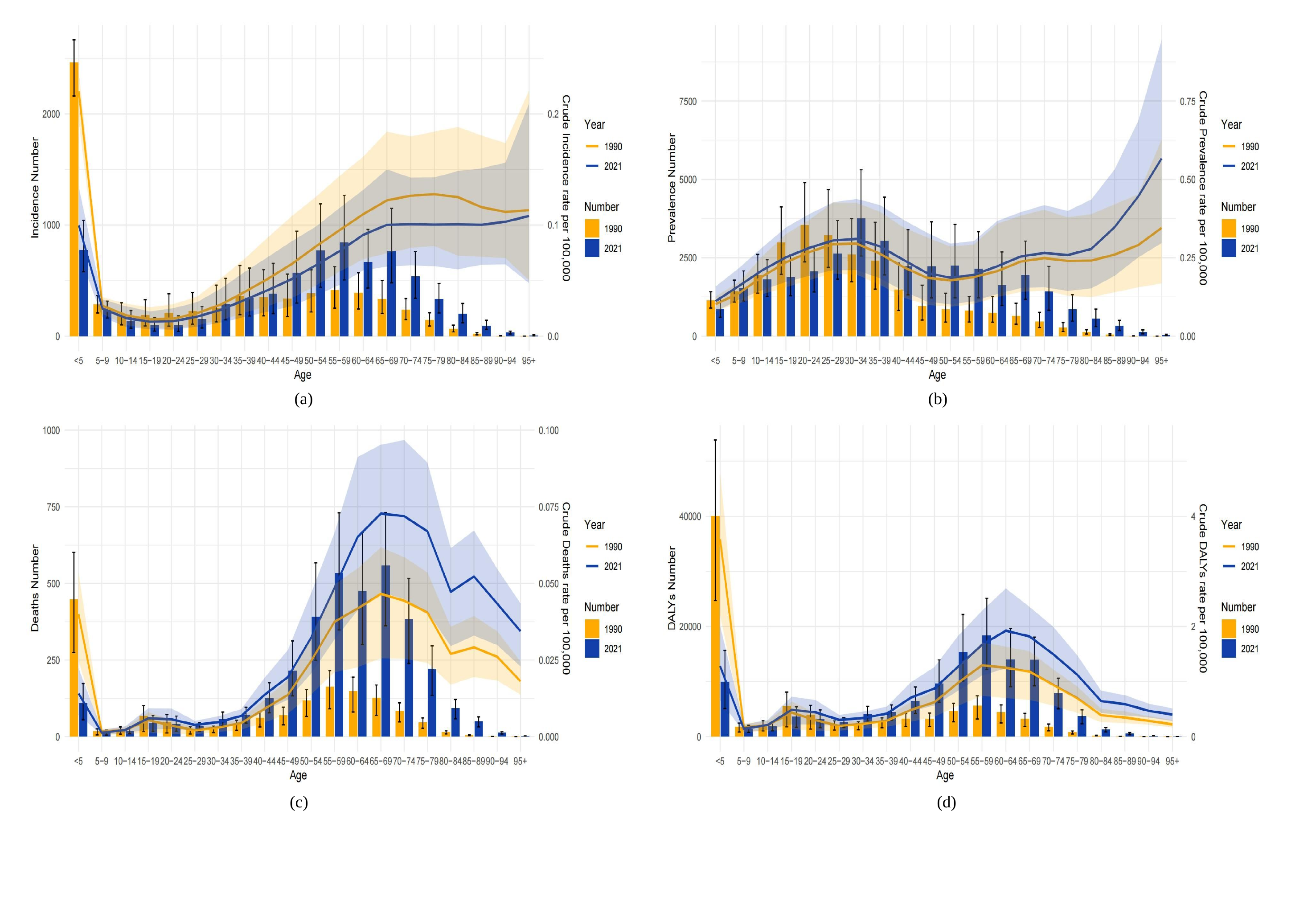

(a)
(b)
(c)
(d)

## Slide 11
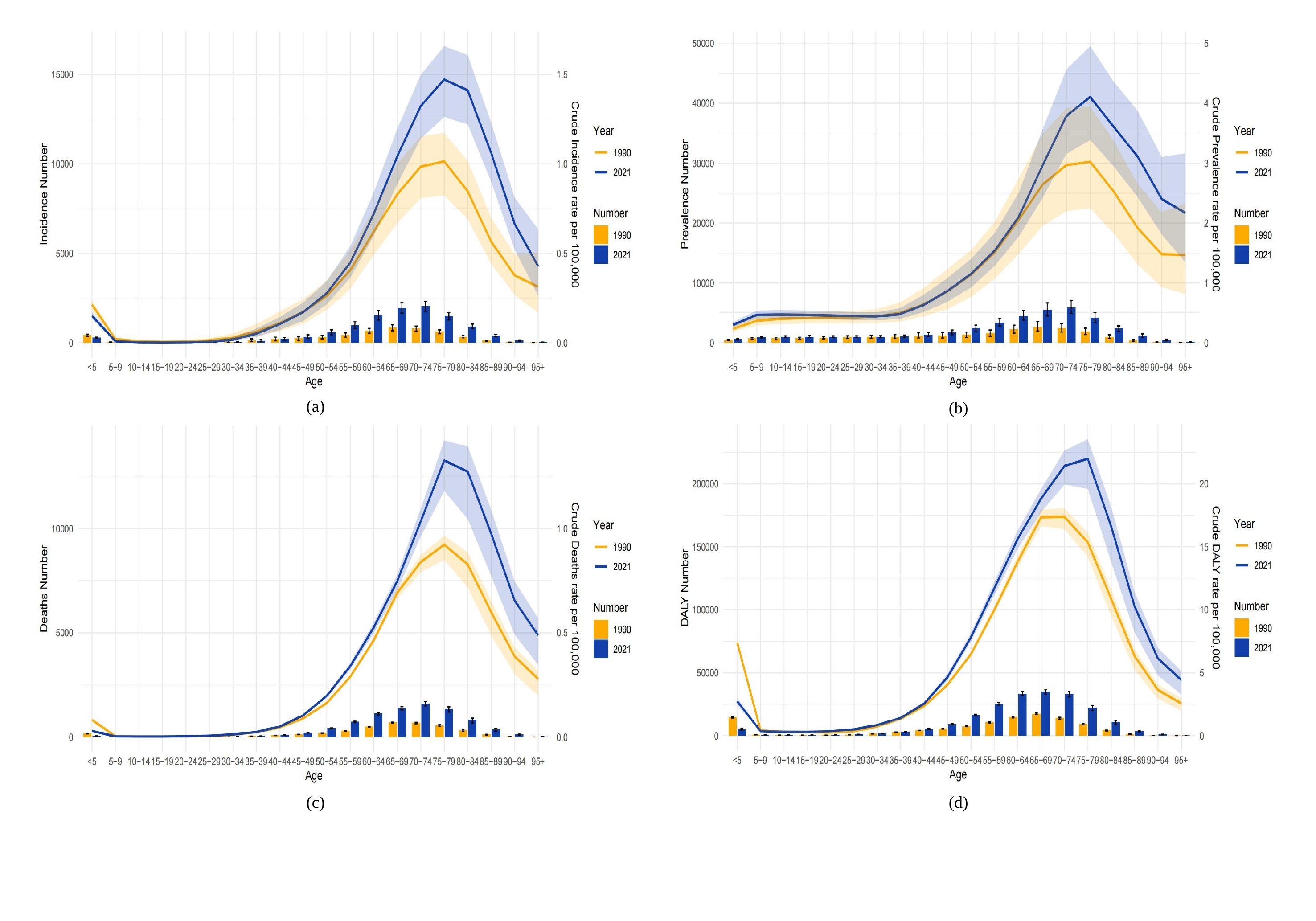

(a)
(b)
(c)
(d)

## Slide 12
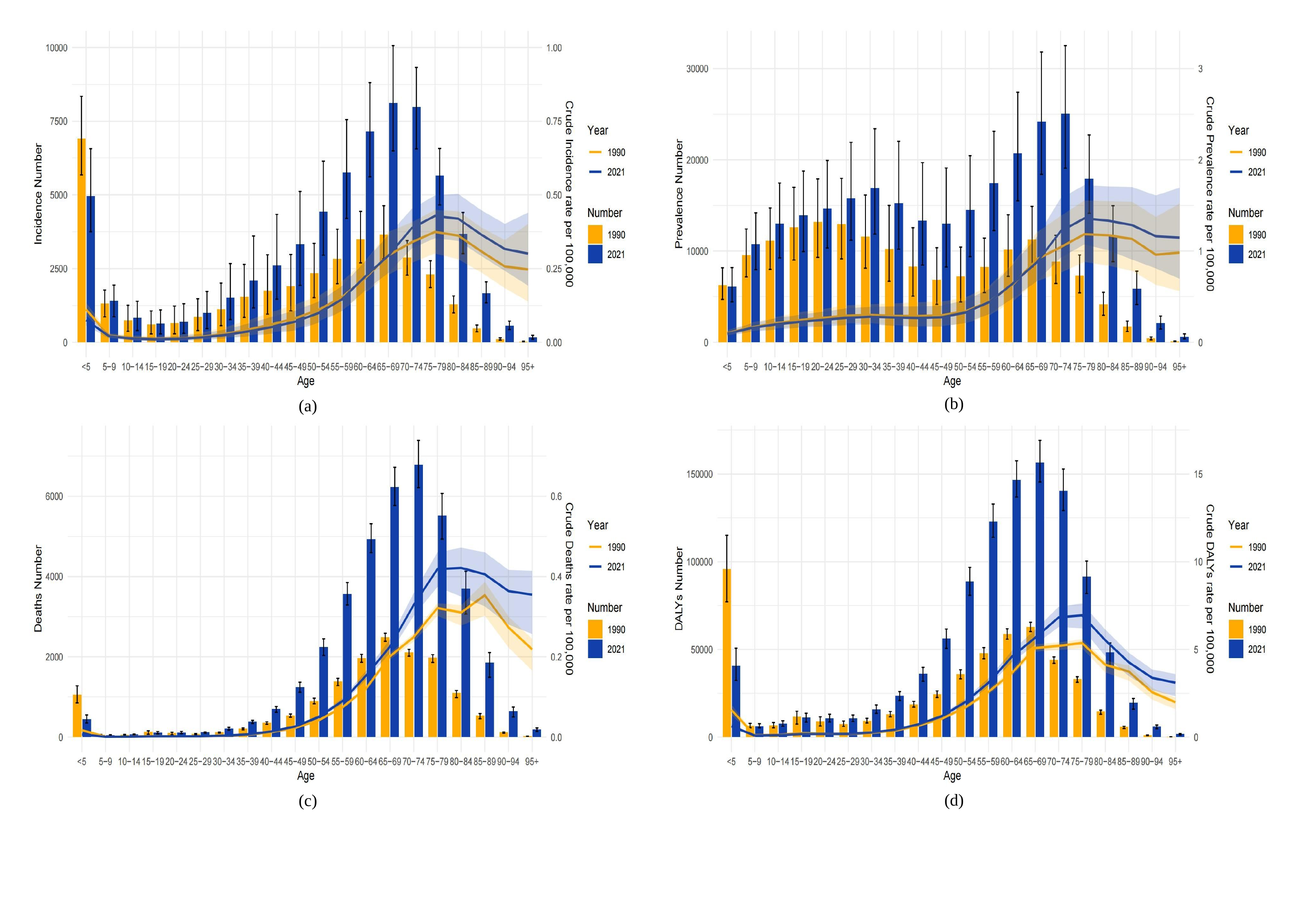

(b)
(a)
(d)
(c)

## Slide 13
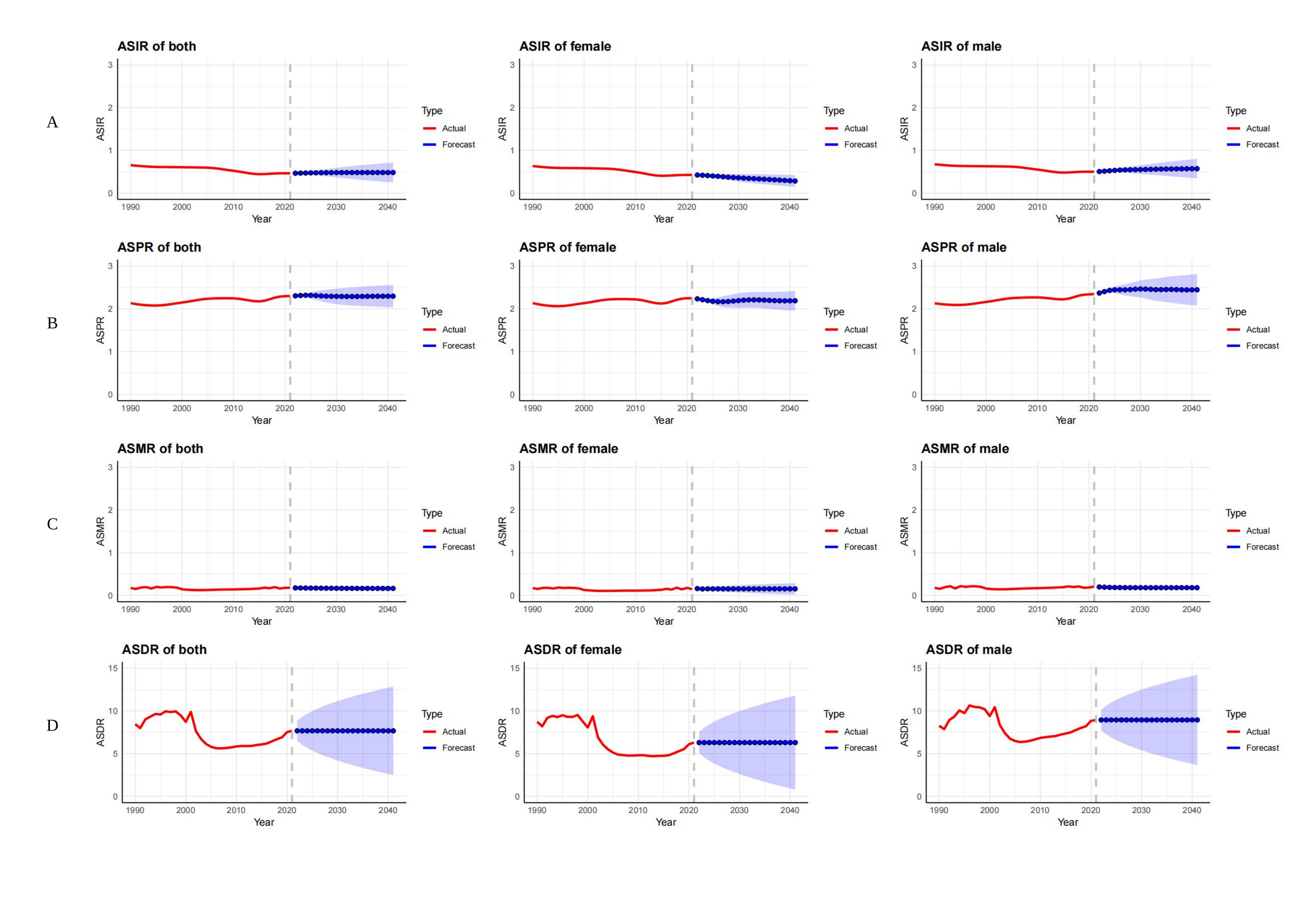

A
B
C
D

## Slide 14
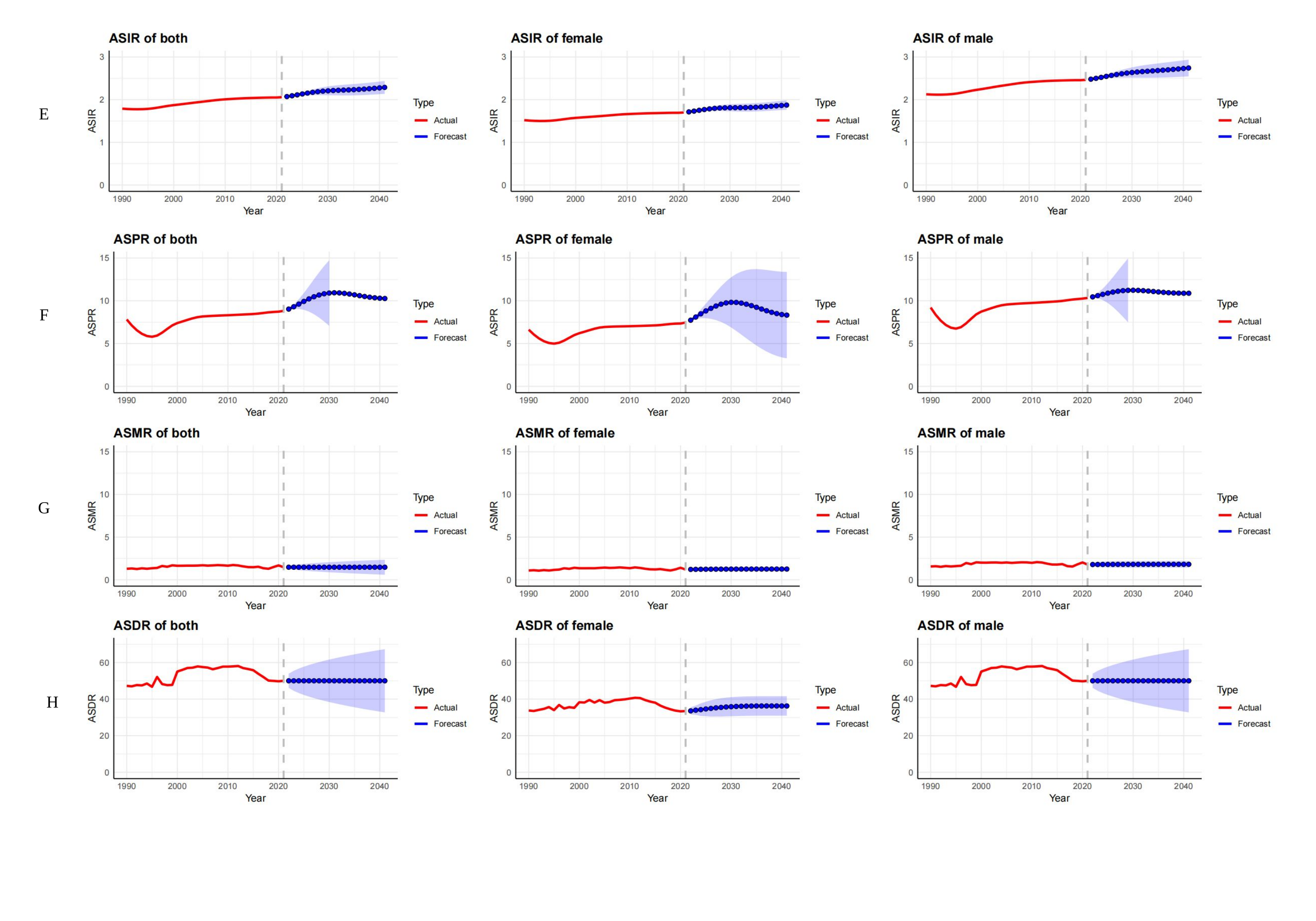

E
F
G
H

## Slide 15
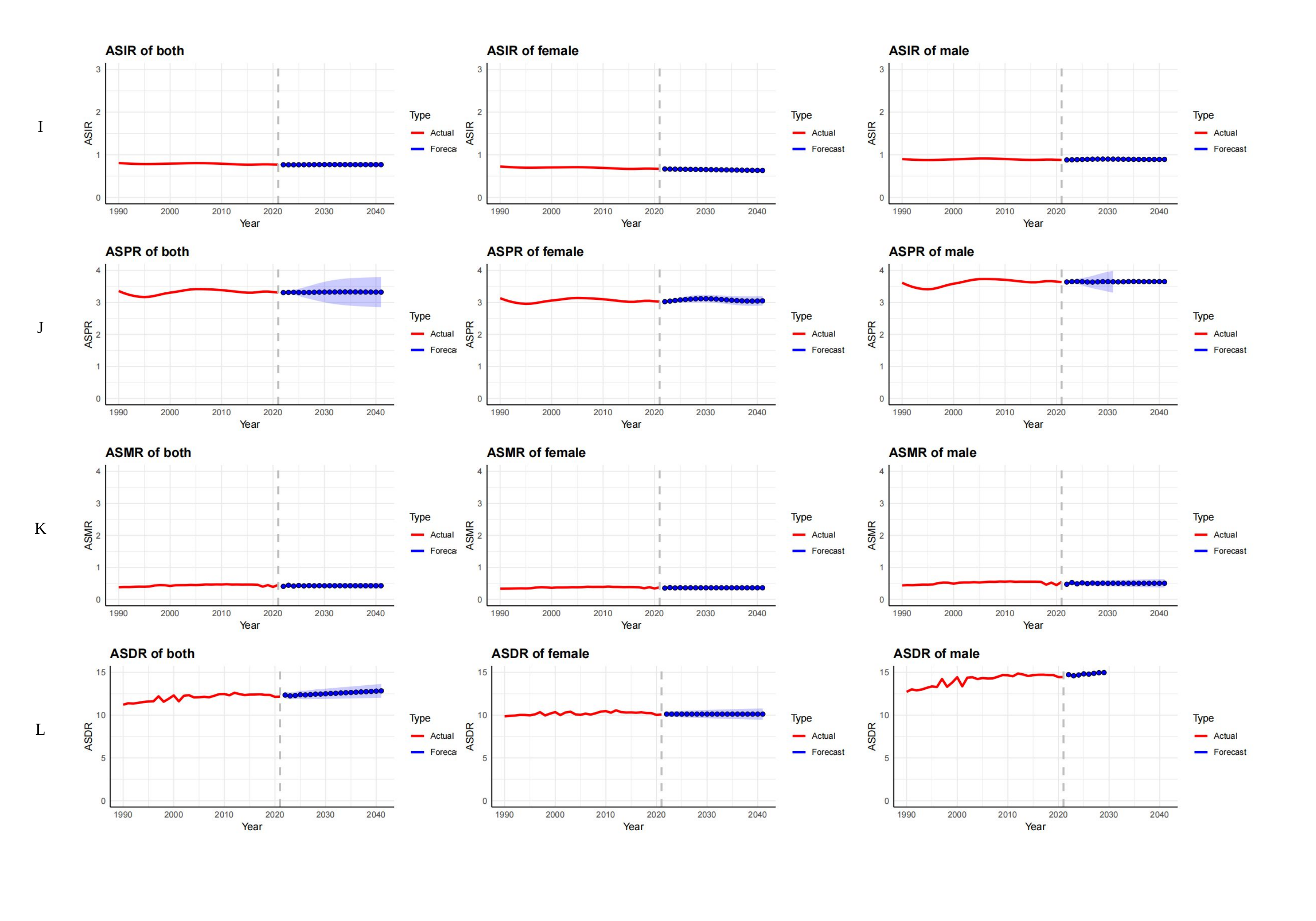

I
J
K
L

## Slide 16
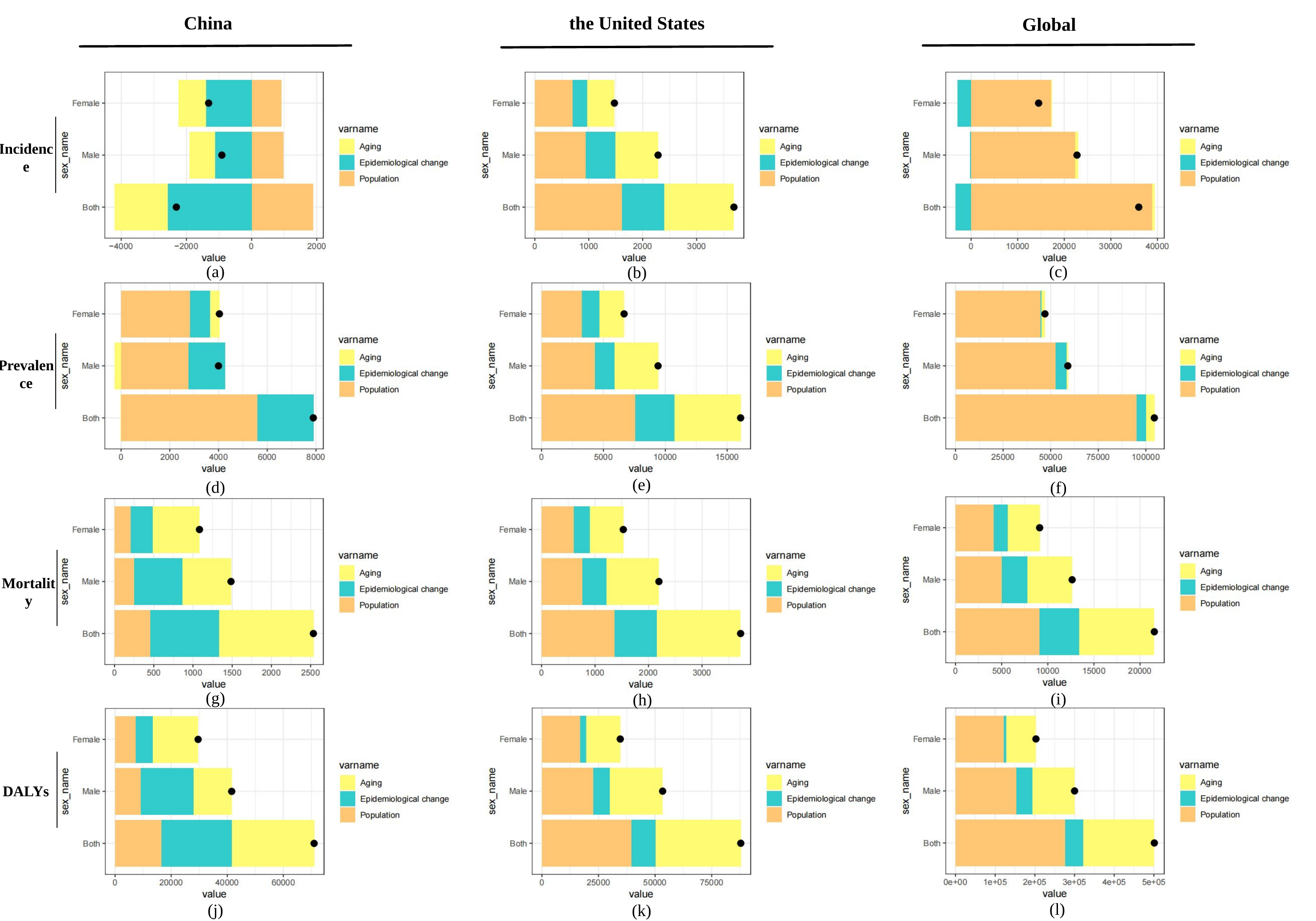

China
the United States
Global
Incidence
(a)
(c)
(b)
Prevalence
(e)
(d)
(f)
Mortality
(g)
(i)
(h)
DALYs
(l)
(j)
(k)
